# Multivalency Controls the Growth and Dynamics of a Biomolecular Condensate

**DOI:** 10.1021/jacs.5c02947

**Published:** 2025-07-08

**Authors:** Julian von Hofe, Jatin Abacousnac, Mechi Chen, Moeka Sasazawa, Ida Javér Kristiansen, Soren Westrey, David G. Grier, Saumya Saurabh

**Affiliations:** † Department of Chemistry, 5894 New York University, New York, New York 10003, United States; ‡ Department of Physics and Center for Soft Matter Research, 5894 New York University, New York, New York 10003, United States; § Department of Chemistry, 6612 Carnegie Mellon University, Pittsburgh, Pennsylvania 15213, United States

## Abstract

Biomolecular condensates are essential for cellular organization and function, yet understanding how chemical and physical factors govern their formation and dynamics has been limited by a lack of noninvasive measurement techniques. Conventional microscopy methods often rely on fluorescent labeling and substrate immobilization, which can perturb the intrinsic properties of condensates. To overcome these challenges, we apply label-free, contact-free holographic video microscopy to study the behavior of a condensate-forming protein in vitro. This technique enables rapid, high-throughput, and precise measurements of individual condensate diameters and refractive indexes, providing unprecedented insight into size distributions and dense-phase macromolecular concentrations over time. Using this method, we investigate the kinetics of droplet growth, aging, and equilibrium dynamics in the model condensate-forming protein PopZ. By systematically varying the concentration and valence of cations, we uncover how multivalent ions influence condensate organization and dynamics, a hypothesis we further test using super-resolution microscopy. Our findings reveal that PopZ droplet growth deviates from classical models such as Smoluchowski coalescence and Ostwald ripening. Instead, we show that condensate growth is consistent with gelation at the critical overlap concentration. Holographic microscopy offers significant advantages over traditional techniques, such as differential interference contrast microscopy, delivering reproducible measurements and capturing condensate dynamics with unparalleled precision. This work highlights the power of holographic microscopy to probe the material properties and mechanistic underpinnings of biomolecular condensates, paving the way for deeper insights into their roles in synthetic systems.

## Graphical abstract

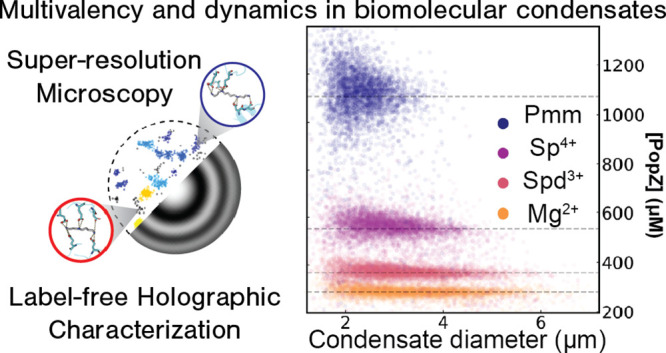

## Introduction

Biomolecular condensates are viscoelastic structures that form through phase separation of associative biological polymers.^1^ They have attracted a great deal of attention in biology because of their ability to create spatial organization within cells without requiring membrane-bound structures,^2,3^ in medicine for their role in physiology and disease,^4^ in biophysics for their potential relevance to the origin of life,^5^ and in chemistry for their emerging applications as self-organized catalytic centers.^6,7^ Condensates can greatly increase the efficiency of the biochemical processes they encapsulate and enable cells to regulate the associated metabolic pathways, which are crucial for signal transduction, gene expression, enzyme activity and warding off environmental stresses.^8,9^

Reconstituting condensates in minimal systems has proven to be invaluable for discovering their physicochemical properties and the biological phenotypes that are associated with them.^3,10,11^ Condensates form by partitioning macromolecules such as proteins and nucleic acids into droplets of high macromolecular concentration dispersed within a dilute phase. The concentration of macromolecules in the dense phase determines the droplets’ viscoelastic and interfacial properties and so influences their morphology.^12,13^ The dense-phase concentration also determines the biological role of the condensate by establishing the droplets’ ability to localize other macromolecules under conditions that support their biochemical activity. The size distribution of condensate droplets offers complementary insights into the mechanisms by which the condensed phase nucleates and grows within a macromolecular solution, and therefore offers insights into the tunability of the droplets’ functionality.^14^

Imaging-based studies of condensate concentration, size distribution and morphology typically rely on fluorescent labeling and often require droplets to be immobilized on glass substrates. Dyes and substrates, however, can perturb the phase behavior of macromolecular solutions as well as the kinetics of droplet condensation. Label-free methods such as quantitative phase imaging^15^ can be used to estimate macromolecular concentration,^16^ but still require droplets to be immobilized on surfaces. Adhesion to a substrate alters the droplets’ apparent size distribution both by distorting individual droplets and also by mediating droplet fusion.^17^ Scanning a surface to build statistics also limits throughput.

We overcome the limitations of conventional analytical techniques by using holographic video microscopy^18,19^ to analyze large populations of unlabeled biomolecular condensates in vitro. Each hologram of a micrometer-scale droplet encodes a wealth of information, including the droplet’s size, shape and refractive index. This information can be retrieved rapidly and with great precision by fitting the droplet’s hologram to a generative model based on the Lorenz-Mie theory of light scattering.^18,20^ Large populations of droplets and particles can be characterized by streaming fluid dispersions through the observation volume of a holographic microscope in a microfluidic channel^19^ and analyzing their holograms in real time.^21^ Holographic characterization yields precise estimates for the size and refractive index of each particle in a precisely specified measurement volume,^22^ and therefore yields exceptionally accurate estimates for the distributions of those quantities.^23^ A particle’s refractive index, in turn, can be interpreted with effective-medium theory to obtain quantitative insights into its composition.^24,25^ Holographic characterization is free from perturbations due to labeling or substrate interactions and can amass data on thousands of particles in a matter of minutes.

Here, we apply holographic microscopy to study the assembly and growth mechanisms of PopZ, a model condensate-forming protein that regulates the cell cycle in the aquatic and soil bacterium, .^3,26,27^ We use holographically measured refractive indexes to infer the concentration of PopZ within individual condensate droplets and thereby to determine how the dense-phase concentration depends on the concentration and valence of the cations used to trigger phase separation. The precision and speed afforded by digital holography enable us to monitor the kinetics of condensate formation, growth and aging over time. These measurements reveal that for divalent cation-mediated condensate formation, the mean droplet diameter grows more slowly than expected for Ostwald ripening, the variance in diameter increases faster than predicted by Smoluchowski kinetics, and at late times, the growth rate aligns with self-regulated aggregation kinetics. While the interplay between ion partitioning, interfacial electric potentials,^28^ and condensate viscosity and polarity^29^ has been elucidated, the influence of ions on condensate substructures and protein mobility remains elusive. Holograms of PopZ condensates reveal that their compositional heterogeneity and dynamics are controlled by the multivalency of cations used, a hypothesis that we tested via super-resolution microscopy and molecular dynamics simulations. In casting new light on macromolecular condensation in a powerful model system, our results also highlight the value of holographic video microscopy for high-throughput and perturbation-free measurements of biomolecular condensates.

## Results and Discussion

### Precise, Substrate-Free Characterization of Condensate Properties

PopZ is an intrinsically disordered protein localized at the poles in that plays a crucial role in localizing and spatially organizing multiple regulatory proteins from the cytoplasm. The structure of PopZ includes an amphipathic α-helix near the N-terminal, which mediates interactions with its “guest” proteins.^30^ PopZ also contains an intrinsically disordered region (IDR) enriched with acidic residues (Figure 1a) that has been suggested to control condensate fluidity,^31^ and C-terminal helical domains crucial for higher-order oligomerization.^30,32^ PopZ undergoes phase separation in vivo^27,31,33^ where it forms condensates that exclude ribosomes and are essential for proper cell division and fitness. Owing to its highly negatively charged IDR, PopZ can be induced to form condensate droplets in vitro in the presence of cations, where it constitutes a versatile model system for studying macromolecular condensation.^3,31^ Figure 1a schematically illustrates the protocol for inducing liquid–liquid phase separation in PopZ solutions by adding magnesium chloride (abbreviated as Mg^2+^, hereafter for simplicity). This produced a population of micrometer-scale condensate droplets observable with differential interference contrast (DIC) microscopy (Figures 1a and S1a).

**Figure 1:**
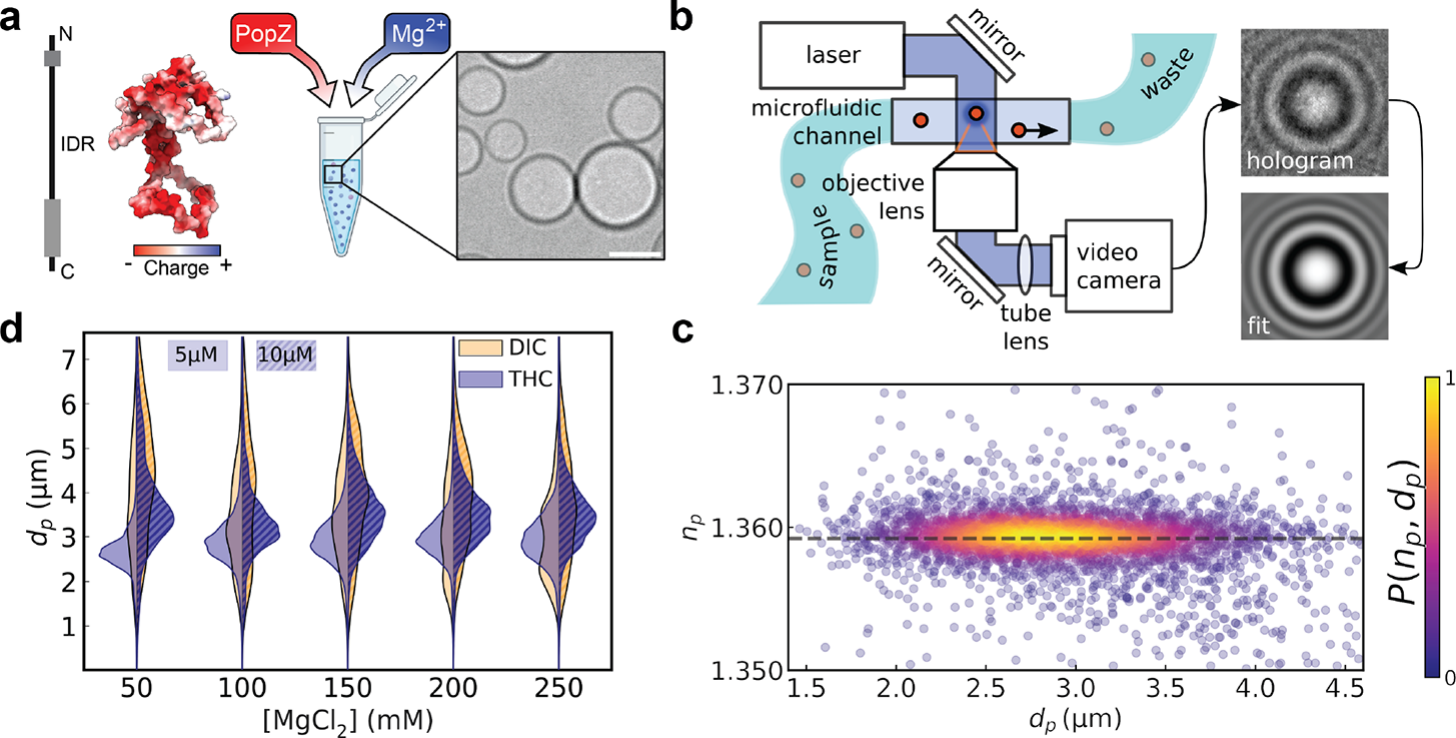
Characterization of PopZ condensates using holographic microscopy. (a) Left: Structural features highlighting the intrinsically disordered region (IDR) and N- and C-terminal alpha helices (gray). The charge distribution of residues across the protein are shown. Right: schematic of PopZ protein condensate formation in the presence of magnesium ions (Mg^2+^), shown alongside a DIC microscopy image of PopZ condensates. The image highlights their spherical morphology. Scale bar: 5 μm. (b) Diagram of the holographic characterization setup for condensate analysis, which employs a microfluidic channel to flow samples through a laser-based holographic microscope. The objective lens captures the scattered light to produce holograms that are subsequently analyzed to extract physical parameters, including diameter *d*_p_, and refractive index, *n*_p_. (c) The scatter plot illustrates the measured *n*_p_ and *d*_p_ values for 4383 condensate droplets, with colors representing the probability density, *P*(*n*_p_,*d*_p_). The horizontal dashed line indicates the mean refractive index, *n*_p_ = 1.35921 ± 0.00003. (d) Violin plots of PopZ condensate size distributions across a range of Mg^2+^ concentrations (50 and 250 μM) at two initial PopZ concentrations (5 and 10 μM). Holographic microscopy provides consistent size measurements without substrate effects, outperforming traditional DIC microscopy.

To analyze PopZ condensates via holographic microscopy, 30 μL of the phase-separated sample was transferred to a microfluidic channel as illustrated schematically in Figure 1b, and the population of droplets passing through the channel in a pressure-driven flow was analyzed. The flow transports particles through the microscope’s laser beam, ensuring that each particle has an opportunity to scatter a proportion of the light. The scattered light interferes with the remainder of the laser beam, and the intensity of the magnified interference pattern constitutes a hologram of the particle that is recorded with a video camera. Provided the number density of condensates does not exceed 10^7^ mL^–1^, holograms of individual droplets are well separated in the field of view and can be analyzed with a generative model^18,20^ based on the Lorenz–Mie theory of light scattering^34^ (Figure 1b) to obtain estimates for the droplet’s diameter, *d*_p_, and refractive index, *n*_p_.

Figure 1c presents holographic characterization results from 4383 PopZ condensates, with the diameter and refractive index of each droplet being represented by a single point. Points are colored by the relative density of observations, *P*(*n*_p_,*d*_p_). The droplets in this sample have a broad distribution of diameters, ranging from *d*_p_ = 1 to 5 μm. By contrast, they have a remarkably narrow distribution of refractive indexes, *n*_p_ = 1.35921 ± 0.00003, which is noteworthy because the refractive index serves as a proxy for the concentration of protein in each droplet.^24,25,35^ Holograms were measured with laser light at a vacuum wavelength of λ = 450 nm, for which the refractive index of the aqueous buffer medium is *n*_m_ = 1.340 ± 0.0001 at room temperature. The droplets’ refractive index therefore is clearly resolved. For such samples, holographic microscopy has been shown^25,36^ to have a detection efficiency close to unity for particles ranging in size from *d*_p_ = 0.5 μm to *d*_p_ = 10 μm. The distribution plotted in Figure 1c therefore provides a comprehensive view of the population of droplets in this size range. The data for this measurement were acquired in the initial 2 min of an 8 min measurement window and therefore represent a snapshot of the sample’s properties. Sequences of such measurements therefore can be used to track the time evolution of the condensate droplets’ properties.

The narrowness of the refractive-index distribution and the lack of correlation between refractive index and size suggests that PopZ is distributed uniformly within each droplet and that the droplets all have the same concentration of PopZ, independent of size. This differs markedly from previously reported holographic characterization results for protein aggregates, whose inhomogeneous fractal structure yields a broad distribution of refractive indexes and a characteristic dependence of refractive index on cluster size.^25,35,37^ Neither the droplets’ sizes nor their refractive indexes are correlated with their height in the microfluidic channel (Figure S1b), furthermore, indicating that hydrodynamic shear has no effect on PopZ condensates’ size and shape under these conditions.^21^

Whereas holographic microscopy can characterize free-flowing condensates, standard techniques such as DIC microscopy require droplets to be immobilized on substrates, which can affect their measured properties (Figure S1c). We quantified the reproducibility of size measurements between DIC and holographic techniques by measuring the Jensen-Shannon divergence (JSD)^38^ between measurement runs (Figure S1d). Holographic size measurements yielded a JSD score of 0.02 ± 0.01, which is an order of magnitude better than the score of 0.40 ± 0.04 obtained for DIC measurements. Size distributions obtained with holographic microscopy therefore are significantly more reproducible, run-to-run. For nonviscoelastic reference particles such as silica beads, DIC and holographic microscopy produce highly consistent size distributions (Figure S1e,f). This result confirms that droplet-to-droplet and run-to-run variations in DIC measurements of PopZ condensates originate from the influence of adhesion to the immobilizing substrate and viscoelastic nature.

### Measurement of PopZ Concentration in the Condensed Phase

Macromolecular condensation is an example of liquid–liquid phase separation mediated by intermolecular interactions.^39^ Because PopZ has an abundance of acidic residues, the phase behavior of its solutions and the properties of its condensates should depend on the concentration and valence of the cations in the buffer. Accordingly, a phase diagram of PopZ in the presence of a range of Mg^2+^ concentrations revealed the presence of a phase boundary for cation concentrations greater than 50 mM (Figure S1a). To systematically assess the effects of Mg^2+^ concentrations in the range from 50 to 250 mM on PopZ condensates, we employed three complementary imaging approaches: DIC microscopy to measure droplet size, confocal fluorescence microscopy to estimate dense-phase concentration, and holographic microscopy to obtain both size and refractive index, which we convert to dense-phase protein concentration. This multimodal strategy allowed us to benchmark traditional methods against the label-free precision of holography. DIC microscopy reveals the presence of condensates at each concentration (Figure 2a), although no differences in condensate protein concentrations can be discerned from the relative intensities of the recorded images. Fluorescent labeling is commonly used to estimate protein concentrations in the dense phase of biomolecular condensates. Accordingly, we used *N*-hydroxysuccinimide (NHS) ester conjugates of BODIPY-FL and JF646 dyes and analyzed the fluorescence intensities inside (*F*_in_) and outside (*F*_out_) condensates using confocal microscopy (Figure S2a–c). The results reveal significant dye-dependent differences, with JF646 showing higher fluorescence intensity in the dense phase compared to BODIPY-FL, even under identical labeling conditions. While the fluorescent signal from BODIPY-FL labeled PopZ increases both inside and outside the condensates, JF646 conjugated PopZ does not show significant differences in the dense phase concentration of PopZ. For the BODIPY-FL conjugated PopZ, *F*_in_/*F*_out_ values increase with Mg^2+^ concentration, suggesting that higher ionic strength promotes labeled-protein sequestration in the dense phase (Figure S2d). However, inconsistencies in *F*_out_ between the dyes and across conditions complicate the interpretation, highlighting how the dyes’ chemical properties influence protein partitioning.

**Figure 2:**
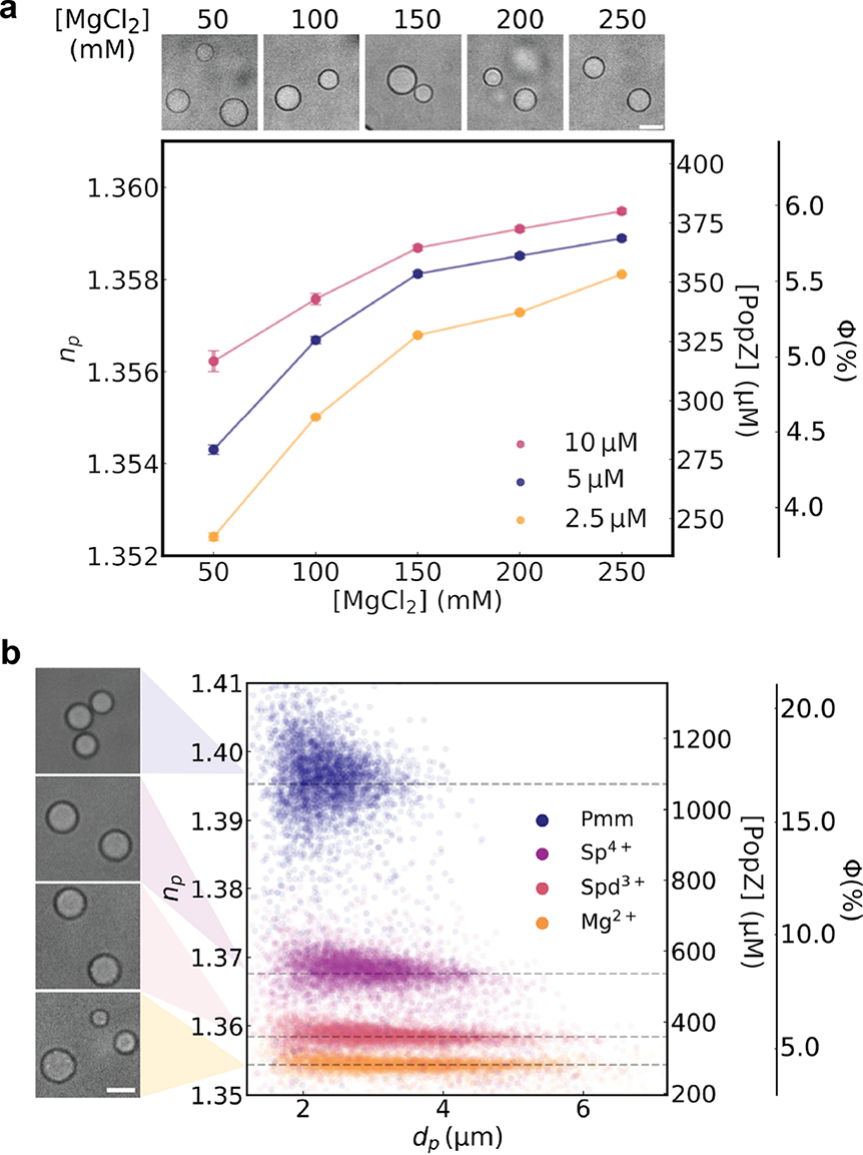
Effect of multivalent ions on the refractive index and size of PopZ condensates. (a) Top: DIC microscopy images of PopZ condensates formed at different Mg^2+^ concentrations (50, 100, 150, 200, and 250 mM). Scale bar: 5 μm. Bottom: Dependence of droplet refractive index (*n*_p_) on Mg^2+^ concentration for different PopZ concentrations (2.5, 5, and 10 μM). Error bars show standard deviations in refractive index. The secondary axes map the refractive index values onto the dense phase concentration and volume fraction (ϕ), respectively, using effective medium theory according to eq 1 in the Supporting Information. (b) Left: DIC microscopy images of PopZ condensates formed by (from top to bottom) Pmm (50 μM), Sp^4+^ (25 mM), Spd^3+^ (33.3 mM), or Mg^2+^ (50 mM). Scale bar: 3 μm. Right: Scatter plot of condensate droplets’ diameters (*d*_p_) and refractive indexes (*n*_p_) colored by the polycation used to trigger condensation. The secondary axes map the refractive index values onto the dense-phase concentration and volume fraction, using effective medium theory. Horizontal dashed lines correspond to the mean refractive index values for each ion type.

Unlike DIC and confocal microscopy, holographic characterization directly measures the refractive indexes of individual condensate droplets. The Supplementary Text details how the refractive index is used to compute the protein volume fraction and absolute concentration in the dense phase. The basis for this precise high-throughput label-free measurement technique is illustrated in Figure S3a. Each point in Figure 2a represents the mean refractive index from a thousand-droplet distribution such as the example in Figure 1c and is reproduced in triplicate. Error bars denote the standard error in the mean for the three samples. The data in Figure 2a reveal that the concentration of PopZ in the dense phase increases both with increasing concentration of PopZ in the starting solution and also with increasing concentration of Mg^2+^. The dense-phase concentration appears to approach a plateau at higher Mg^2+^ concentrations, suggesting that the divalent ion and PopZ exhibit saturable binding mediated via specific interactions. The plateau concentration appears to depend comparatively weakly on the overall concentration of PopZ in the system. The influence of Mg^2+^ concentration on the measured protein concentration in the dense phase suggests a role for cation valence in modulating the composition of the condensed phase.

We hypothesized that increasing the valence of the cation used to trigger phase separation would further increase the dense phase concentration. To test this idea, we form PopZ condensates in the presence of polyvalent cations with different valence: trivalent spermidine trichloride (Spd^3+^), tetravalent spermine tetrachloride (Sp^4+^), and a polyamidoamine dendrimer called PAMAM (Pmm) that has 16 terminal amines.^40,41^ PopZ condensates are formed at pH 7.0. Under these conditions, the polyamines Spd^3+^ and Sp^4+^ are fully protonated and thus exhibit cationic valences of +3 and +4, respectively.^42^ Although the degree of protonation of Pmm at pH 7.0 is not precisely known,^41^ this dendrimer should have an effective valence greater than that of Sp^4+^, and thus serves as an extreme case of cationic valence. To facilitate comparison, Mg^2+^, Spd^3+^, and Sp^4+^ are added at concentrations such that they result in an equivalent total positive charge. The DIC images in Figure 2b illustrate that all four types of cations induce liquid–liquid phase separation and produce spherical condensate droplets. The data in Figure 2b reveal that droplets are produced in roughly the same numbers with all four types of cations but that their protein content varies dramatically with cation valence.

From divalent Mg^2+^ to polyvalent Pmm, PopZ condensates undergo a 4-fold increase in their concentration. To place this in context, the volume fraction of PopZ in the dense phase increases from ϕ = 0.04 in the presence of Mg^2+^ to ϕ = 0.16 in the presence of Pmm (Figure S3b). For reference, the phase diagram of PopZ as a function of each polyamine’s concentration is provided in Figure S4. The measured volume fraction of protein in PopZ condensates is substantially lower than the volume fraction of polymers in typical latex colloids,^43^ suggesting that PopZ condensates are likely to be much softer.^44^ Biologically, this softness may enable PopZ condensates to remain mechanically compliant and highly dynamic, properties that could facilitate efficient molecular exchange, spatial remodeling, and responsiveness to cell cycle or stress-related signals. Finally, the distribution of refractive index values also broadens with higher cation valence, suggesting that polyvalent cations create more opportunities for structural heterogeneity within each droplet, a hypothesis we test later. The increase in dense-phase concentration is associated with a decrease in the average droplet diameter and a decrease in the dispersity in size. When combined with the observation that droplets form at roughly the same number density in all four systems, this result suggests that increasing cation valence does not substantially affect the rate of droplet nucleation, but subsequently fosters growth of smaller denser condensate droplets. Accordingly, we investigated next the growth mechanisms of PopZ condensates through time-resolved holographic imaging.

### Measured Condensate Size Distributions Constrain Models for Droplet Growth

The ability of cells to control the size of condensate droplets has been proposed as a mechanism to modulate the macromolecular concentration within the droplets and thereby to regulate the droplets’ functionality.^8^ Monitoring the size of condensate droplets can also be useful for diagnosing diseased states. For example, dysregulation of the sizes of nucleolar condensates has been correlated with cancer prognosis severity.^45^ A recent study proposes that condensate sizes in biological systems follow either an exponential distribution or a power-law distribution depending on the relative rates of nucleation and coalescence.^14^ These findings, consistent across native cellular condensates, synthetic optogenetic systems, and computer simulations, suggest a biophysical principle governing condensate formation and size regulation in cells.

To gain insight into the growth mechanisms of PopZ condensates, we use the capabilities of holographic microscopy to monitor the size distribution of condensate droplets as a function of time after phase separation is triggered by the addition of Mg^2+^. Due to the resolution limit of holographic microscopy, only droplets larger than approximately 500 nm in diameter can be reliably detected.

Typical results for the time-dependent size distribution over the detected population, *P*(*d*_p_,*t*), are plotted in Figure 3a. Smaller particles are likely to be present, particularly in the dilute phase, but we currently have no basis for assessing their number or size distribution. Quantitative analysis contraindicates droplet coalescence and Ostwald ripening as viable explanations for the observed time evolution of *P*(*d*_p_,*t*), and instead suggests that these droplets grow by self-regulating coagulation of macroions at the critical gel point.

**Figure 3:**
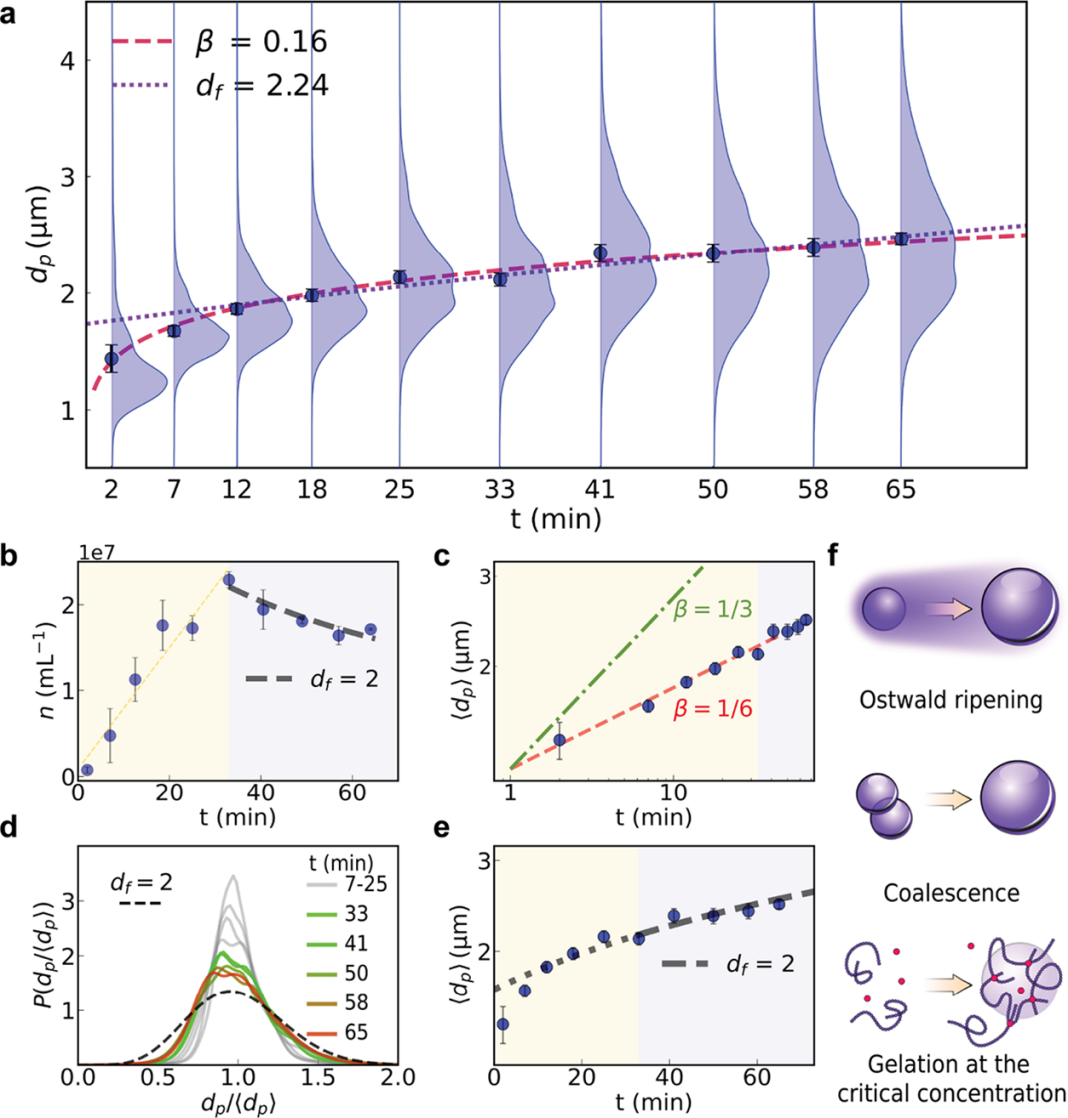
Time-dependent evolution of PopZ condensates. (a) Violin plots showing condensate size distributions, *P*(*d*_p_,*t*), for 2.5 μM PopZ at 50 mM Mg^2+^, at various time points, demonstrating the progressive increase in condensate size distribution over time. Discrete (dark blue) points represent the mean condensate diameter, ⟨*d*_p_(*t*)⟩, at each sampling time, with error bars indicating the standard error of the mean at each time. The red dashed curve is a fit to a power law, ⟨*d*_p_(*t*)⟩ ∼ *t* ^β^. (b) Droplet concentration over time, following predictions from self-regulated kinetics at late times. The initial increase in concentration suggests nucleation-driven processes. (c) Log–log plot of the mean droplet diameter showing power-law scaling. The fit exponent, β = 0.16 ± 0.01, is inconsistent with the value of 1/3 expected for Ostwald ripening (green dot-dashed line). (d) Probability densities, *P*(*d*_p_/⟨*d*_p_⟩), of droplet diameters scaled by the mean droplet diameter at each sampling time, *t*. These distribution functions should collapse onto a single curve (black dashed line) for a system displaying dynamic scaling characteristic of growth by droplet coalescence. (e) Semilog plot of the mean droplet diameter. At late times, the evolution of mean droplet diameter aligns with eq 3 consistent with self-regulated kinetics (black dashed line). (f) Schematic representations of the three growth mechanisms discussed: Ostwald ripening, coalescence, and gelation at the critical concentration.

The mean droplet diameter at each measured time interval, ⟨*d*_p_(*t*)⟩, is denoted by discrete points in Figure 3a, with error bars representing the standard error in the mean. These averages are computed only over droplets detected within the resolution limits of holographic microscopy (diameters > 500 nm). The dashed curve passing through these points suggests that the observed droplets grow as a power law in time,

$$\langle d_{p}(t)\rangle ∼t^{\beta }$$ 1

with the exponent β = 0.16 ± 0.01. Power-law growth is consistent with coarsening by coalescence, which is described by the Smoluchowski kinetic model.^46,47^ Assuming that droplets collide and merge at a rate that is limited by diffusion, the Smoluchowski equation predicts that their size distribution should have the form^48^

$$P(d_{p},t)=\frac{2W(\alpha )}{\Gamma (\alpha +1)}{\left(W(\alpha )\frac{d_{p}}{\left\langle d_{p}(t)\right\rangle }\right)}^{2\alpha +1}\exp\left(-W^{2}(\alpha )\frac{d_{p}^{2}}{\langle d_{p}(t)\rangle^{2}}\right)$$ 2a

The width of this distribution,

$$W\left(\alpha \right)=\left(\alpha +1\right)\frac{\Gamma \left(\alpha +\frac{3}{2}\right)}{\Gamma \left(\alpha +2\right)}$$ 2b

is set by the size dependence of the droplets’ diffusion coefficient,

$$D(d_{p})∼d_{p}^{-\alpha }$$ 2c

In the dynamic scaling hypothesis, the exponent α establishes the droplets’ growth rate:

$$\beta =\frac{1}{2\left(\alpha +1\right)}$$ 2d

The observed value of β suggests α = 1.94 ± 0.10 which differs significantly from the standard Stokes result, α = 1, for solid spheres, but could be consistent with the scaling predicted for a fractal polymer gel.^49^

Even if coalescence can account for the growth in the mean droplet diameter, it does not explain the observed broadening of the size distribution. Eq 2a suggests that *P*(*d*_p_,*t*) should collapse onto a single master curve if *d*_p_ is normalized by ⟨*d*_p_(*t*)⟩. Instead, the scaled distribution functions plotted in Figure 3d broaden with time, and only appears to reach an asymptotic form after 45 min. This broadening is apparent because holographic microscopy builds a statistical sample rapidly enough to resolve it. The asymptotic distribution width corresponds to α = 3.8 ± 0.1, which differs from the dynamic scaling hypothesis and does not have an obvious interpretation in terms of the droplets’ microstructure or dynamics. This disagreement effectively rules out droplet coalescence as the dominant coarsening mechanism.

Ostwald ripening also causes coarsening with power-law time dependence.^50^ In this case, however, the Lifshitz–Slyozov–Wagner theory predicts the growth exponent to be β = 1/3, which is twice the observed value. The difference is clearly resolved in Figure 3c. Droplet growth therefore does not appear to be driven by simple diffusive exchange of macromolecules between liquid-like droplets. This is consistent with previous studies of PopZ condensates using fluorescence recovery after photobleaching in vitro^3^ and lower mobility fractions in vivo.^31^ The apparent absence of Ostwald ripening in this system suggests that the surface tension of PopZ condensates must be low, which also appears to be the case for condensates of coiled-coil proteins.^51^

Holographic microscopy provides accurate values for the number density of condensates, *n*(*t*), throughout the accessible size range^23^ and therefore tracks changes in droplet number due to processes such as nucleation and coalescence independent of changes in their observable size distribution. The data in Figure 3b show that the number density of detectable condensates increases for the first half hour after condensation is triggered, suggesting that nucleation continues for several minutes after condensation is triggered. The number density declines after half an hour, which suggests that coarsening outstrips the nucleation rate thereafter.

The observed growth exponent, β ∼ 1/6 is reminiscent of the slow coagulation of macroions in the presence of multivalent counterions,^52^ which is a model system for the coagulation of protein chains in the presence of polyvalent cations. As condensate aggregates grow, the Coulomb barrier between them increases, reducing the likelihood of further coagulation. This “self-regulated” growth mechanism, which begins at time *t*_0_ > 0, predicts a logarithmic growth law for the mean droplet diameter

$$\langle d_{p}(t)\rangle =d_{0}\log\left(\frac{t-t_{0}}{t_{0}d_{f}}+e\right)$$ 3

where *d*_0_ represents the mean droplet size at time *t*_0_. The model further suggests that the concentration of typical aggregates decreases logarithmically with time, following

$$\langle d_{p}(t)\rangle =d_{0}{\left(\frac{n_{0}}{n(t)}\right)}^{1/d_{f}}$$ 4

where *n*_0_ is the concentration of droplets at time *t*_0_, and *n*(*t*) is the time-dependent concentration. To test this model, we measured the droplet concentration over time, and identified two distinct regimes (Figure 3b). At early times, the concentration increases monotonically, consistent with nucleation driven growth. Around the 30 min mark, the concentration begins to decrease steadily. In this later regime, we fit the concentration data to eq 4 assuming a fractal dimension *d*_f_ = 2. Furthermore, the logarithmic dependence of the mean droplet size, as shown in Figure 3d, is consistent with this fractal dimension. Together, these observations suggest that at late times, the slow growth of condensates follows the predictions of self-regulated kinetics, rather than Ostwald ripening or Smoluchowski coagulation. The fractal dimension of the condensates in this regime matches that of a cross-linking polymer at the gel point (Figure 3f), reinforcing the idea of a self-regulated growth model.

### Physicochemical Perturbations Drive Condensates Out of Equilibrium

Reconstituted condensates are usually studied at or near equilibrium. Living systems, by contrast, rely on condensates’ properties far from equilibrium. To bridge this gap, we combine DIC and holographic microscopy to study how condensates respond to changes in temperature and the chemical environment. PopZ condensates imaged via DIC microscopy appeared smaller and less distinct at higher temperatures (Figure 4a), consistent with an upper critical solution temperature (UCST). Upon cooling back to 30 °C, condensates reappeared with similar size and number density as before heating, indicating that this temperature-induced dissolution and condensation process is fully reversible (Figure S4f). Holographic analyses of the same condensates revealed tight size and refractive index distributions of condensates at 30 and 42 °C. This was also different from DIC data, where we observed smaller condensates at 42 °C, potentially a surface attachment artifact. However, at 50 and 60 °C, we observed a skewed distribution containing a low refractive index population of large condensates and a high refractive index population of small condensates. The former population was not observed via DIC microscopy, potentially due to longer settling times and low particle density. Specifically, the number density of the detected condensates dropped from 1.6 × 10^7^ mL^–1^ at 30 °C to 9.5 × 10^5^ mL^–1^ at 60 °C, which is consistent with the idea that PopZ condensates exhibit UCST behavior.^3^

**Figure 4:**
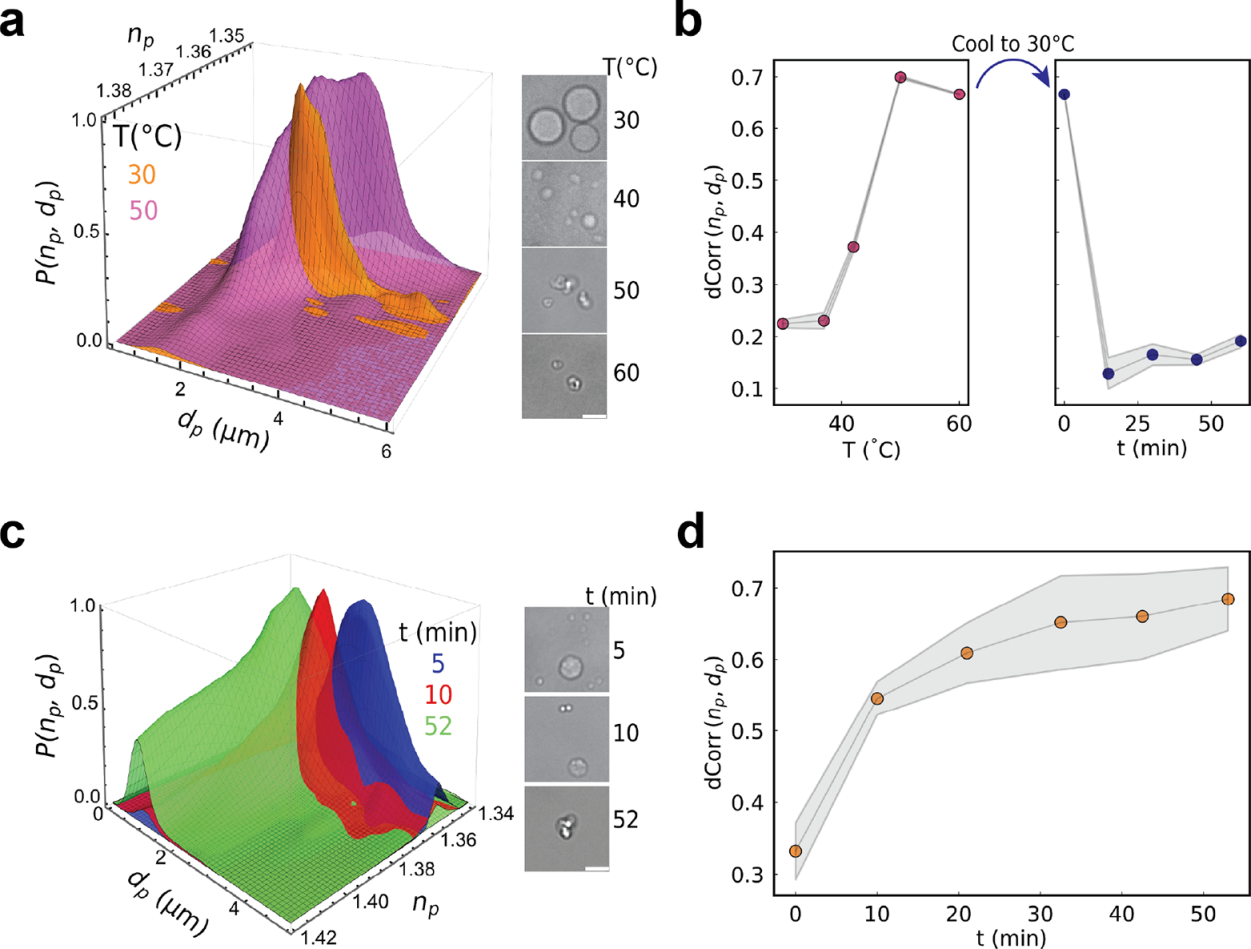
Out of equilibrium behavior of PopZ condensates. (a) Left panel: Surface plot of the refractive index (*n*_p_) against the diameter (*d*_p_) of the same condensates at two different temperatures (30 and 50 °C), with probability densities *P*(*n*_p_,*d*_p_) shown as the heights of the surfaces. At the two highest temperatures, the shapes of the *n*_p_ v *d*_p_ distributions are typical of condensates far from equilibrium. At lower temperatures, refractive indexes converge to a specific dense phase concentration value. Right panel: DIC microscopy images of PopZ condensates (5 μm at 150 mM Mg^2+^) at different temperatures (30, 42, 50, and 60 °C), showing morphological changes with increasing temperature. Scale bar: 3 μm. (b) Distance correlation between refractive index and diameter dCorr­(*n*_p_,*d*_p_) acts as a proxy for distance from equilibrium as a function of temperature, showing an increase at higher temperatures. Right panel: sharp reversal of the temperature-induced increase in dCorr­(*n*_p_,*d*_p_) by returning the system to 30 °C, demonstrating a time-dependent recovery of dCorr­(*n*_p_,*d*_p_) over 50 min. Shaded areas represent errors obtained by bootstrapping. (c) Left panel: Surface plot of the refractive index (*n*_p_) against the diameter (*d*_p_) of the same condensates. Right panel: DIC microscopy images of PopZ condensates as a function of time post lipoic acid addition. Scale bar: 3 μm. (d) Time-dependent behavior of the dCorr­(*n*_p_,*d*_p_) as a function of time, before, and after lipoic acid addition, showing a sharp increase in dCorr­(*n*_p_,*d*_p_) just after addition of lipoic acid followed by a gradual stabilization over time. Shaded areas represent error bars, computed by combining uncertainties from two sets of measurements.

We used distance correlation, as defined in the Supplementary Text, as a proxy for the distance of the system from equilibrium. Condensates far from equilibrium typically appear asymmetrical, as shown in the DIC micrographs in Figure 4a. Such morphologies exhibit strong anticorrelations between *n*_p_ and *d*_p_, which are captured by measuring the distance correlation dCorr­(*n*_p_,*d*_p_). Large values of dCorr­(*n*_p_,*d*_p_) correspond to larger deviations from equilibrium. During the heating process dCorr­(*n*_p_,*d*_p_) obtained from size distributions increased monotonically while upon cooling back to 30 °C, dCorr­(*n*_p_,*d*_p_) converged to lower values as the condensates approached equilibrium (Figure 4b). Next, we used lipoic acid, a compound previously shown to fluidize PopZ condensates^3^ as a chemical perturbation. We monitored the condensate dissolution process as a function of time after adding 5 mM lipoic acid (Figure 4c). In this case, we observed the distribution shift toward small-sized condensates with high refractive indexes. This distribution could result from fluidization of large condensates, resulting in the formation of smaller, protein-rich assemblies that may be stabilized by hydrophobic interactions with lipoic acid. This interpretation is further strengthened by the observed increase in dCorr­(*n*_p_,*d*_p_) immediately after the addition of lipoic acid, followed by a moderate increase as the system reaches its new equilibrium (Figure 4d). Taken together, these data show the utility of holographic characterization, and provide a transferable analytical framework to study biomolecular condensates away from equilibrium.

### Excipient Multivalency Modulates Condensate Substructure and Dynamics

Building on the observation that polyvalent cations promote denser PopZ condensates and broaden the refractive index distribution (Figure 2b), we next investigated the structural heterogeneity within individual droplets and its relationship to condensate dynamics. The hypothesis generated by holographic microscopy, that polyvalent cations introduce greater opportunities for structural heterogeneity was tested by examining both the spatial organization and temporal behavior of PopZ molecules within condensates. We applied single-molecule localization microscopy (SMLM) on condensates that were sparsely labeled with JF646-conjugated PopZ.^53^ By employing oblique illumination to further minimize perturbations, we observed individual fluorescent molecules within the condensates, which after localization and reconstruction, revealed distinct subdiffraction clusters, as shown in Figure 5a (top). The degree of clustering varied depending on the cation used, with more prominent clustering observed in Mg^2+^ and Pmm samples. We applied a density-based spatial clustering of applications with noise (DBSCAN) analysis to the single molecule data to assess the number of localizations per cluster.^54^ DBSCAN analyses show that clusters inside condensates formed with Mg^2+^ and Pmm have a higher number of localizations compared to Spd^3+^ and Sp^4+^. In addition to nanoscale clusters, we detected a population of rapidly moving molecules, particularly in samples containing Spd^3+^ and Sp^4+^, which could not be precisely localized due to their fast motion in and out of the focal plane.

**Figure 5:**
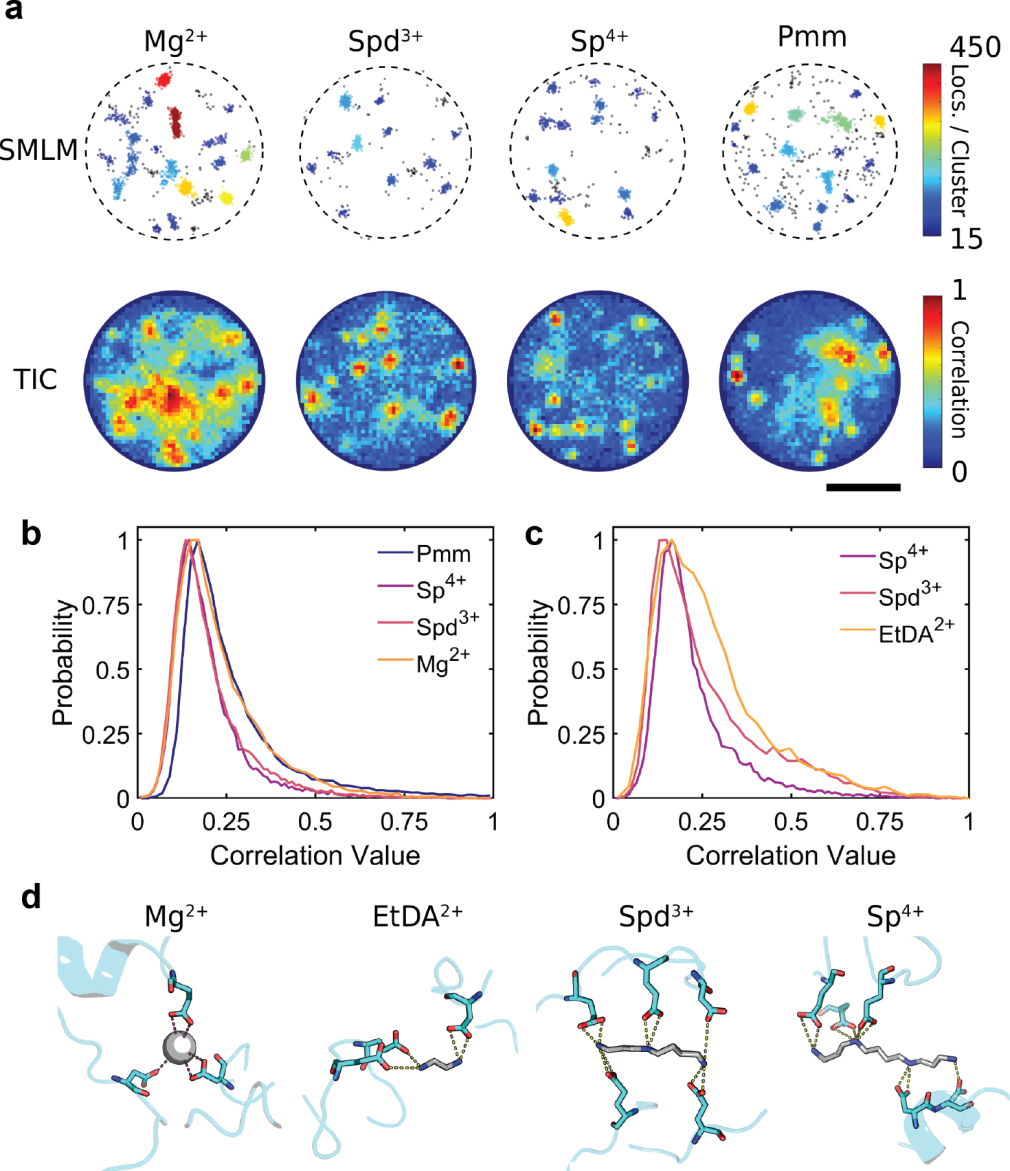
Effects of multivalent ions on PopZ condensate structure and dynamics. (a) Top: Single molecule localization microscopy and bottom: temporal image correlation analyses of PopZ condensates in the presence of various cations: Mg^2+^, Spd^3+^, Sp^4+^, and Pmm dendrimer. The condensates were labeled using 0.001% (v/v) JF646-conjugated PopZ. SMLM highlights clustered localizations colored by the number of localizations in the cluster and outliers colored in black, while TIC maps regions of correlated molecular localizations within condensates. Scale bar: 2 μm. (b) Normalized frequency distributions of TIC correlation values for condensates formed with different multivalent ions. Comparison of Mg^2+^, Spd^3+^, Sp^4+^, and Pmm reveals distinct shifts in molecular motion dynamics. (c) Comparison of EtDA^2+^, Spd^3+^, and Sp^4+^ at matched ionic strengths indicates decreased temporal correlation with higher valence of the multivalent cations. (d) Molecular interaction models showing representative binding configurations of PopZ with Mg^2+^ (gray sphere), EtDA^2+^, Sp^4+^, and Spd^3+^. Green dashed lines indicate hydrogen bonding and ionic interactions between PopZ and the multivalent cations.

Rapid motion of labeled PopZ molecules led to fluctuations in the fluorescent background within the condensates that were analyzed via temporal image correlation spectroscopy (TICS).^55^ Temporal correlation over a single time lag (20 ms) allowed us to differentiate dynamic from static regions, with blue regions in Figure 5a (bottom) indicating higher molecular dynamics (low temporal correlation) and red regions representing static areas (high temporal correlation). Static regions within Spd^3+^ and Sp^4+^ correlated well with clusters of high density of molecules. In contrast, condensates formed using Mg^2+^ and Pmm showed some clusters that were dynamic despite having a high molecular density as measured via DBSCAN analyses (Figure S5a). This TICS analysis revealed that condensates formed with Sp^4+^ exhibited the highest molecular dynamics, followed by Spd^3+^ and Pmm, while Mg^2+^ displayed the lowest dynamics (Figure 5b). This ranking is based on the spatial distribution of pixel-wise temporal correlation values, where lower correlation (blue regions) indicates higher local molecular motion. However, this comparison was confounded by differences in ionic strength between the samples and the dendrimeric structure of Pmm, which makes direct interpretation challenging. To address this, we conducted a second experiment where the ionic strength was matched across all samples, and the valence of the multivalent cations was systematically varied, progressing from ethylenediamine dichloride (EtDA^2+^) to Spd^3+^ and Sp^4+^. In this experiment where the ionic strengths were controlled (Figure 5c), EtDA^2+^ showed the lowest dynamics, with a broad and right-shifted correlation value distribution, followed by Spd^3+^ and Sp^4+^, which exhibited narrower distributions, indicative of greater internal dynamics. This trend becomes clear when looking at correlation values of the three cations between 0.25 and 0.5. Additionally, correlations of these systems were tested using 5 to 20 lag times. At 20 lag times, correlation distributions of EtDA^2+^ (Figure S5b) and Spd^3+^ (Figure S5c) shifted to slightly lower values while remaining relatively constant with Sp^4+^ (Figure S5c). Taken together, these results demonstrate that increased cation multivalency enhances molecular dynamics within PopZ condensates and sustains these dynamics over longer time scales, highlighting the critical role of excipient valency in tuning the internal dynamic environment of biomolecular condensates.

To further explore the molecular basis of these observations, we performed all-atom molecular dynamics (MD) simulations of PopZ in the presence of Mg^2+^, EtDA^2+^, Spd^3+^, and Sp^4+^. The simulations revealed distinct interaction profiles (Figure 5d). Mg^2+^ exhibited a tightly coordinated network of electrostatic interactions with acidic residues within a small volume, while larger, multivalent molecules like Sp^4+^ showed multiple, spatially distributed hydrogen-bonding interactions between amino hydrogens and acidic residues across the molecule.

Further, an analysis of the number of molecular interactions observed between each cation and PopZ chains shows that Mg^2+^ exhibits the highest interaction counts across all bond numbers, reflecting its strong and frequent interactions with protein residues (Figure S6). In contrast, Sp^4+^, while having the lowest interaction counts for 1 and 2 bonds, dominates at higher bond numbers (5 to 6 bonds) due to its ability to form multiple simultaneous interactions. Among the multivalent ions, shorter chains like EtDA^2+^ show higher interaction counts at lower bond numbers, while longer chains such as Sp^4+^ and Spd^3+^ are better at higher bond numbers due to their extended structures and additional interaction sites. The distributed hydrogen bonding, lower frequency of molecular interactions, and lower interaction strength of hydrogen bonds compared to electrostatic interactions offered via Mg^2+^ likely explain the enhanced dynamics and compositional heterogeneity observed in condensates formed with higher valence excipients.

Our findings reveal that the valence of excipients used to trigger condensation is a critical determinant of structural heterogeneity and molecular dynamics in biomolecular condensates. By promoting distributed interactions within the condensate, higher valence cations create more opportunities for dynamic rearrangements. This insight into the role of multivalency in driving condensate behavior provides a deeper understanding of the physicochemical principles underlying condensate formation and regulation, with implications for material properties in biological and synthetic systems.

## Conclusions

Measuring the precise size and composition of biomolecular condensates is essential to understand their formation, regulation, and impact on cellular processes. These measurements help differentiate functional condensates from pathological aggregates and assess bulk-like properties. Traditional methods rely on fluorescence labeling or surface attachment, which can alter condensate properties. Here, we report the use of holographic microscopy to measure the size and concentration of the dense phase of a model bacterial condensate, PopZ. This method is able to collect information for ∼5000 particles in ∼1 min, achieving greater precision and accuracy than conventional methods. Holograms of PopZ condensates revealed that dense phase concentrations are tunable through excipient and protein concentrations. Our results show that the initial protein concentration governs the resultant condensate sizes, recapitulating previous observations in vivo in a PopZ overexpression system.^31^ The detection of bimodal size distributions in PopZ condensates highlights the unique ability of this measurement technique to resolve complex size distributions that other methods fail to detect, uncovering multiple growth mechanisms. While these mechanisms are not fully accounted for in the current model, the model nonetheless captures the essential aspects of condensate growth.

Time-dependent size distributions of PopZ condensates discerned via holography revealed growth dynamics different from previously reported exponential or power-law size distributions of condensates.^14^ We applied a polyelectrolyte-attachment model^52^ that predicts slow condensate growth, which was consistent with observations at late times. Using the distance correlation between index of refraction and size of condensate populations, we investigated the approach to equilibrium for PopZ condensates as well as their dissolution and reformation by temperature and lipoic acid. The data collectively illustrate the dynamic response of PopZ condensates to thermal and chemical stress, highlighting both immediate and reversible changes in their viscoelastic properties. Previous research has demonstrated the effect of electrostatic interactions on the molecular scale by influencing macromolecular structure within a condensate,^56^ but spatial heterogeneity within a condensate remains poorly understood. Guided by our observation of the broadening distribution of *n*_p_ with multivalent excipients, we explored the nanoscale architecture of PopZ using super-resolution microscopy. Correlation analyses highlighted the effect of electrostatic interactions on condensate composition. These results demonstrate that excipient multivalency modulates the structural heterogeneity and molecular dynamics of PopZ condensates by enabling distributed interactions that drive dynamic rearrangements, emphasizing the critical role of cation valence in condensate regulation. Importantly, this study illustrates the utility of holographic microscopy as a powerful hypothesis-generating tool, enabling noninvasive insights into condensate substructure that can be further tested and refined using complementary, minimally perturbative methods such as single-molecule localization and molecular modeling to uncover mechanisms of intracondensate organization in situ.

Despite these advances, the method has limitations and relies on certain assumptions. The existing commercial implementation of holographic microscopy has a lower size limit of 0.5 μm, which means that condensates smaller than this limit are not detected (see Figure 3a). Nevertheless, the high precision offered by the method enables hypothesis generation about condensate composition and viscoelasticity for in vivo condensates that can be followed up using live-cell methods. When performed at a single wavelength, holographic microscopy provides a single value for the refractive index of each particle or droplet. This value must be interpreted with effective-medium theory^24,25,57^ and modeling^58,59^ to infer the composition of a heterogeneous material such as a multicomponent condensate. Future work will focus on studying two- and three-component condensates with the goal of estimating species-specific volume fractions to get relative occupancy of condensate scaffolds. Another area of research will be the application of holographic microscopy to study condensates that regulate enzymes, utilizing the dCorr­(*n*_p_,*d*_p_) parameter to estimate the activity inside the condensate. Finally, comparison between conventional and holographic imaging modalities promises access to the relationship between condensate growth mechanisms and surface tension and therefore paves the way for a careful analysis of size distributions to infer interfacial energies that could be used for assessing condensate viscoelastic properties.

Our findings raise important questions about how the structural and dynamic properties of PopZ condensates observed in vitro relate to their cellular functions. The ionic and thermal perturbations we applied were designed to probe physical mechanisms but also parallel physiologically relevant factors. Many alphaproteobacteria, including *Caulobacter*, accumulate polyamines such as homospermidine and spermidine,^60^ though their role in modulating condensates in vivo remains to be explored. Likewise, our observation of UCST-like behavior under heat stress suggests a possible mechanism for regulating protein localization or proteolytic activity via condensate dissolution. In *Caulobacter*, for instance, the ClpXP protease machinery is localized within PopZ condensate,^61^ raising the possibility of thermal stress-responsive condensate remodeling. Although the condensates studied here are larger than native PopZ foci, their concentration-dependent growth mirrors trends seen in overexpression systems,^31^ suggesting shared physical principles that warrant further testing in vivo. Finally, the principle that multivalent ions modulate nanoscale heterogeneity and internal molecular mobility is likely to apply broadly to biomolecular condensates across diverse cellular systems.

## Supplementary Material



## Notes

Supplementary text on characterization of condensates using holographic imaging, supplementary figures, and experimental methods (PDF)
