# Supplementary material for: Multivalency Controls the Growth and Dynamics of a Biomolecular Condensate
Source: J Am Chem Soc. 2025 Jul 8;147(29):25242–53. doi: 10.1021/jacs.5c02947 (PMC12291466; doi:10.1021/jacs.5c02947)
Supplement: Supplementary file 1 [file ja5c02947_si_001.pdf]

Supporting Information: Multivalency controls the growth and dynamics of a biomolecular condensate

Julian von Hofe,[†,§] Jatin Abacousnac,[‡,§] Mechi Chen,[†] Moeka Sasazawa,[†] Ida Javér Kristiansen,[†] Soren Westrey,[¶] David G. Grier,[‡] and Saumya Saurabh[*,†]

†*Department of Chemistry, New York University, New York, NY, 10003*

‡*Department of Physics and Center for Soft Matter Research, New York University, New York, NY, 10003*

¶*Department of Chemistry, Carnegie Mellon University, Pittsburgh, PA, 15213*

§*These authors contributed equally*

E-mail: saumya@nyu.edu

Contents

Holographic Measurement of Protein Concentration in the Condensed Phase S-2

Jensen-Shannon Divergence S-5

Distance Correlation S-5

Supplementary Figures S-6

Fig. S1: Characterization of PopZ condensates and their phase behavior S-7

Fig. S2: Characterization of PopZ condensates via fluorescence microscopy S-8

Fig. S3: Concentration and volume fraction measurements of PopZ condensates . S-9
Fig. S4: Phase behavior of PopZ with different excipients and temperature S-10
Fig. S5: Temporal image correlation analysis of PopZ condensates with various excipients S-11
Fig. S6: Molecular interactions of PopZ with various excipients observed via MD simulation S-12

Experimental **S-13**

Protein expression and purification S-13
Condensate preparation S-13
Lipoic acid preparation and addition S-13
Differential Interference Contrast (DIC) microscopy sample preparation S-13
Condensate imaging with DIC S-14
Condensate imaging and characterization with holographic microscopy S-15
Dye labeling and confocal imaging of condensates S-16
Single molecule localization microscopy of PopZ condensates S-17
All-atom molecular dynamics simulations of PopZ and multivalent cations S-19

Holographic Measurement of Protein Concentration in the Condensed Phase

Total Holographic Characterization (THC) (the commercial implementation of holographic microscopy with microfluidics) yields a value for the refractive index, n_p, of each condensate droplet with part-per-thousand precision.[S1,S2] This information can be used to infer precise values for the volume fraction and absolute concentration of proteins in the dense phase of a phase-separated solution. This method has not been reported previously and represents a new application area for holographic microscopy.

The measured refractive index is related to the volume fraction, ϕ, of protein in the dense phase through Maxwell Garnett effective-medium theory,[S2–S4]

$$\phi = \frac{L(m_p)}{L(m_0)}, \tag{1a}$$

where $m_p = n_p/n_m$ is droplet's refractive index relative to that of the medium, $m_0 = n_0/n_m$ is the relative refractive index of the protein itself, and

$$L(m) = \frac{m^2 - 1}{m^2 + 2}, \tag{1b}$$

is the Lorentz-Lorenz function. The volume fraction of protein within the condensate is proportional to the protein concentration, c,

$$c = \frac{\phi}{V_c}, \tag{1c}$$

where V_c is the volume of a single protein chain. The refractive index of the medium, n_m, is measured conveniently with an Abbe refractometer (Abbe-3L Refractometer, Fisher Scientific). Measuring the dense-phase protein concentration also requires precise values for the intrinsic refractive index of the protein, n_0, and the associated single-chain volume, V_c.

The intrinsic refractive index of the protein can be obtained from its amino acid sequence using effective-medium theory together with tabulated data[S5] for the refractive indexes, n_j^{D}, and specific volumes, v_j of the pure amino acids. Tabulated values are reported for the a vacuum wavelength of 589 nm, which corresponds to the sodium D line. The result for an N-amino-acid protein,

$$n_0^{\mathrm{D}} = \sqrt{\frac{2L_N^{\mathrm{D}} + 1}{1 - L_N^{\mathrm{D}}}}, \tag{2}$$

is a function of the volume-weighted Lorentz-Lorenz factor,

$$L_N^{\mathrm{D}} = \frac{\sum_{j=1}^N v_j \, L(n_j)}{\sum_j v_j}. \tag{3}$$

This result is scaled for use at other wavelengths, λ, using a standard result for protein refractive-index increments,[S6]

$$n_0(\lambda) = n_0^{\mathrm{D}} \left(0.942 + \frac{20\,000}{\lambda^2} \right). \tag{4}$$

This approach yields $n_0 = 1.689 \pm 0.001$ for the intrinsic refractive index of PopZ at the imaging wavelength used for holographic characterization, $\lambda = 450\,\mathrm{nm}$. This value can be used in Eq. (1a) to convert measurements of droplets' refractive indexes into estimates for the volume fraction of protein within the droplets, as presented in Fig. 2a. The typical single-droplet uncertainty in the estimated volume fraction is $\Delta\phi = \pm 0.2\,\%$, based on propagation of uncertainties in n_0^{D} and the parameters in Eq. (4).[S6]

The largest uncertainty in estimating protein concentrations can be ascribed to the value used for the single-chain volume, V_c. This is the volume associated with the protein's intrinsic light-scattering properties, which is substantially smaller than the volume subtended by the protein's tertiary structure. We obtain an estimate for V_c by using an Abbe refractometer to measure the refractive index, n_s, of bulk protein solutions as a function of protein concentration, c. These values are converted into estimates for the volume fraction of protein in the bulk using the computed value of n_0^{D} and the measured refractive index of the buffer, n_m, as inputs to Eq. (1a) as inputs. Typical results for PopZ are plotted as discrete points in Fig. S3a. The dependence of n_s on c yields an estimate for the optical volume, V_c, through Eq. (1c). The result for PopZ, $V_c = (260 \pm 67)\,\mathrm{nm}^3$, agrees with independent estimates obtained numerically using the CALVADOS model,[S7] which also are plotted in Fig. S3a. We use the calibrated value of V_c to convert holographically measured values of the droplet refractive index into the estimates for the dense-phase PopZ concentration that

are reported in Fig. 2. The same protocol can be used to measure the dense-phase concentration in other protein solutions undergoing phase separation, and can be generalized to measure macromolecular concentrations in heterogeneous condensates.

Jensen-Shannon Divergence

To quantify the reproducibility of size measurements using holographic characterization and DIC, we compute the Jensen-Shannon Divergence (JSD) scores between sets of measurements from the same method. Size distributions between two runs using the same technique provides an estimate for the reproducibility of the method. The probability distributions are most similar when the JSD approaches 0. The JSD itself is derived from the Kullback Leibler Divergence (KLD), which, for probability distributions p_1 and p_2 is given as

$$\mathrm{KLD}(p_1|p_2) = p_1(x) \sum_{x \in \mathcal{X}} \ln \frac{p_1(x)}{p_2(x)}. \tag{5}$$

If m is a mixture distribution,

$$m = \frac{1}{2}(p_1 + p_2), \tag{6}$$

the JSD is computed as[S8]

$$\mathrm{JSD} = \frac{1}{2}\Big[\mathrm{KLD}(p_1|m) + \mathrm{KLD}(p_2|m)\Big]. \tag{7}$$

We computed the JSD scores between holographic microscopy runs and obtained consistently lower scores across the range of Mg^{2+} when compared to the JSD scores between DIC runs. As a benchmark, we compared a probability distribution obtained from holography with a uniform distribution with the same range $[a, b]$, where a and b are the minimum and maximum sizes respectively measured using holographic microscopy.

Distance Correlation

Distance correlation scores between condensates' refractive indexes and sizes were used as a proxy for distance from equilibrium. The distance correlation score is defined as[S9]

$$\mathrm{dCorr}(n_p, d_p) = \frac{\mathrm{dCov}(n_p, d_p)}{\sqrt{\mathrm{dVar}(n_p)\,\mathrm{dVar}(d_p)}}, \tag{8}$$

the distance covariance between n_p and d_p, normalized by the square root of the product of distance variances. The distance covariance between two quantities X and Y is defined as

$$\mathrm{dCov}(X, Y) = \frac{1}{n^2} \sum_{i}^{n} \sum_{i}^{n} D(x_i, x_j) \cdot D(y_i, y_j), \tag{9}$$

where $D(x_i, x_j)$ is the double-centered distance matrix for quantity X of size n, and the distance variance is the covariance with itself

$$\mathrm{dVar}(X) = \mathrm{dCov}(X, X). \tag{10}$$

Unlike the more commonly used Pearson correlation, the distance correlation does not assume a linear relationship between quantities. Additionally, the distance correlation score $\mathrm{dCorr}(n_p, d_p)$ is constrained between 0 and 1, where $\mathrm{dCorr}(n_p, d_p) = 0$ strictly applies only to independent variables.

Supplementary Figures

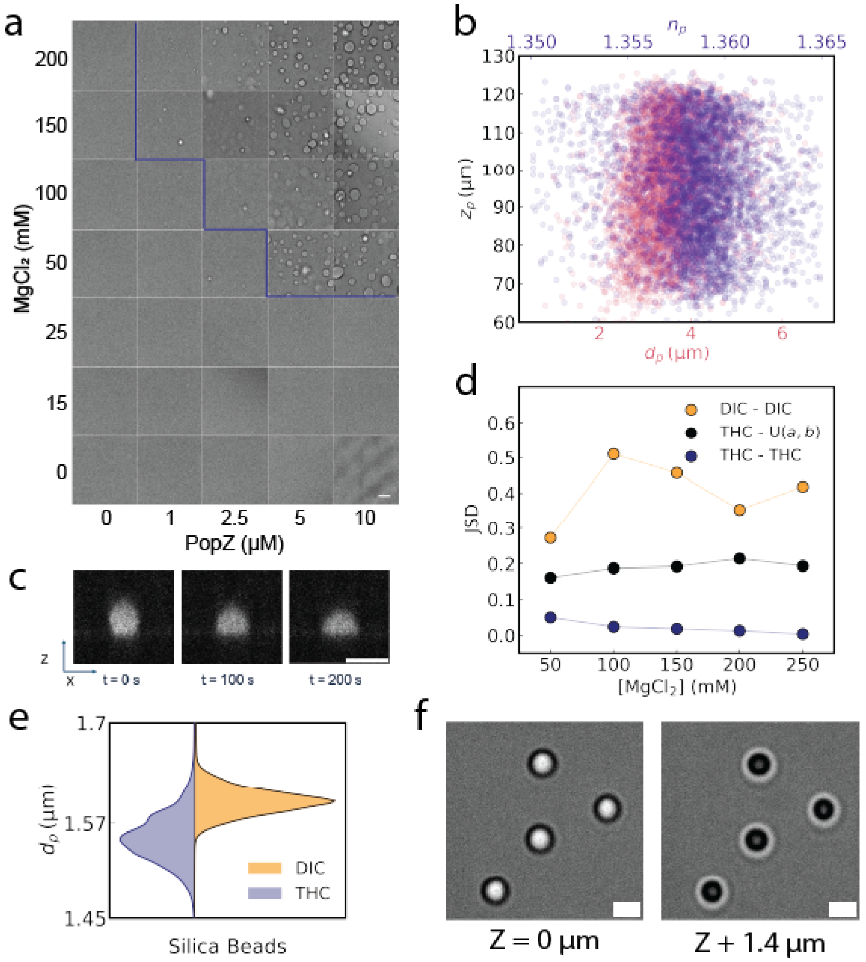

Figure S1: **Characterization of PopZ condensates droplets and their phase behavior.** (a) Phase behavior of PopZ as a function of protein and Mg^{2+} concentration measured via DIC. Scale bar: 5 µm. (b) Scatter plot demonstrating that the measured diameter (d_p) and refractive index (n_p) of individual PopZ condensates is independent of their axial position (z_p). (c) Time-lapse images showing the relaxation dynamics of a single PopZ condensate (labeled using 5 % BODIPY-FL-PopZ) over 200 s on a glass coverslip, visualized in the x-z plane using confocal microscopy. Scale bar: 5 µm. (d) The Jensen-Shannon Divergence (JSD) is computed for the size measurements shown in Figure 1d. Comparing JSD scores between holography measurements and DIC measurements reveals that holography measurements are more reproducible than DIC measurements. The JSD score between a size distribution obtained from holography and one that follows a uniform distribution with the same mean and standard deviation is shown for reference. (e) Size distributions of 1.57 µm silica particles imaged using holographic or DIC microscopy. Holographic size measurements show a broader distribution that more accurately measures the size of the silica particles as compared to DIC. 2507 particles were analyzed from holography and 207 were analyzed from DIC. (f) Representative DIC images of 1.57 µm silica particles either in-focus (Z = 0 µm) or at an optimal height for segmentation and size distribution analysis (Z + 1.4 µm). Scale bar: 2 µm.

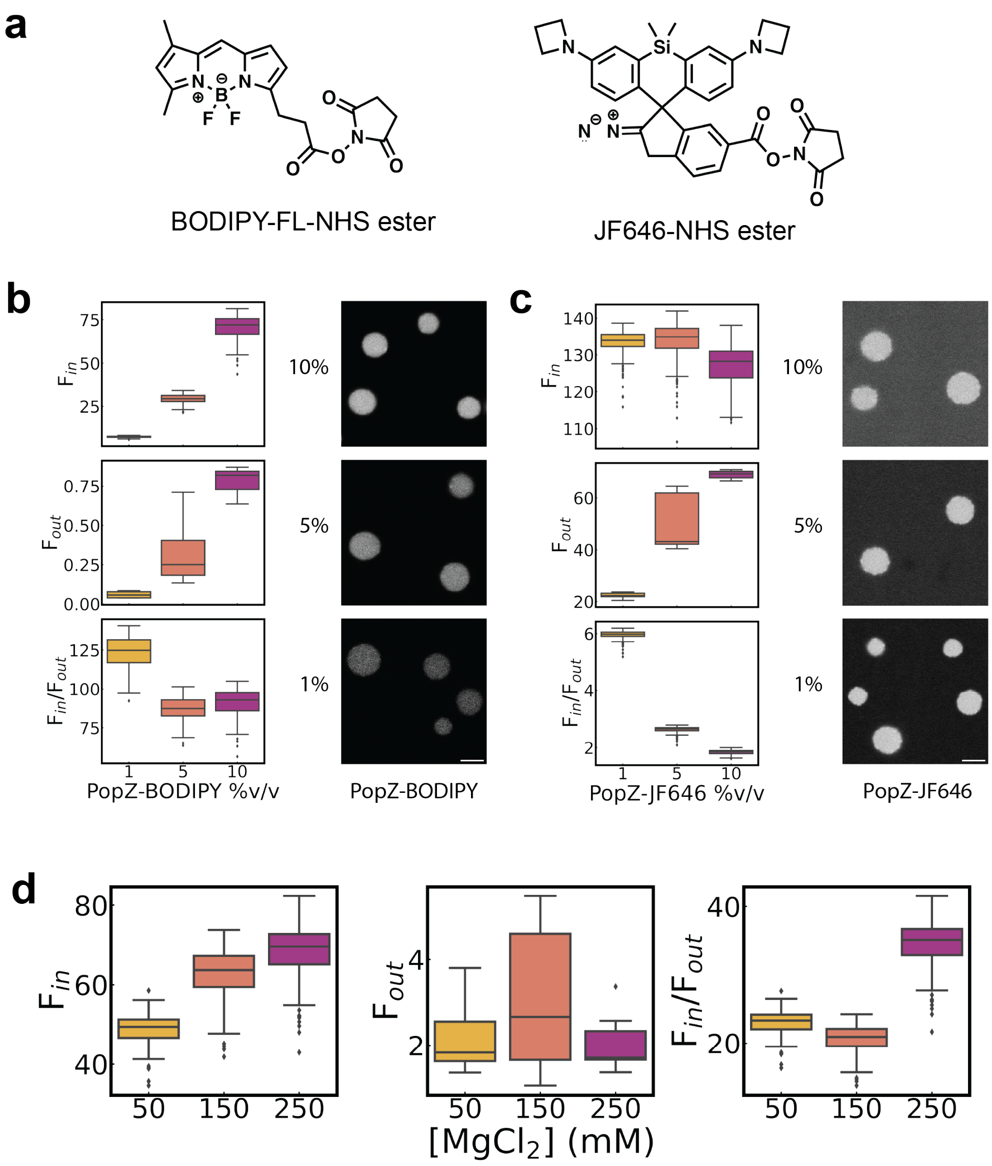

Figure S2: **Characterization of PopZ condensates via fluorescence microscopy.** (a) Structure of the NHS-ester derivatives of BODIPY-FL and JF646, the dyes used in this study. (b) Left panels: Boxplots of fluorescence intensity in (F_{in}) and outside (F_{out}) condensates at different percent v/v additions of BODIPY-FL-labeled PopZ to an unlabeled PopZ sample. 5 µM PopZ and 200 mM Mg^{2+} were prepared for each experiment. Relative concentrations of dense phase were determined using the ratio of $F_{\text{in}}/F_{\text{out}}$. Representative confocal images are shown. (c) Same data as in (b) but with PopZ condensates labeled with PopZ-JF646. Scale bar: 5 µm. (d) Boxplots of F_{in} and F_{out} of PopZ-BODIPY labeled condensates (5% v/v) as a function of Mg^{2+} concentration. Relative concentrations of dense phase PopZ were determined using the ratio of $F_{\text{in}}/F_{\text{out}}$.

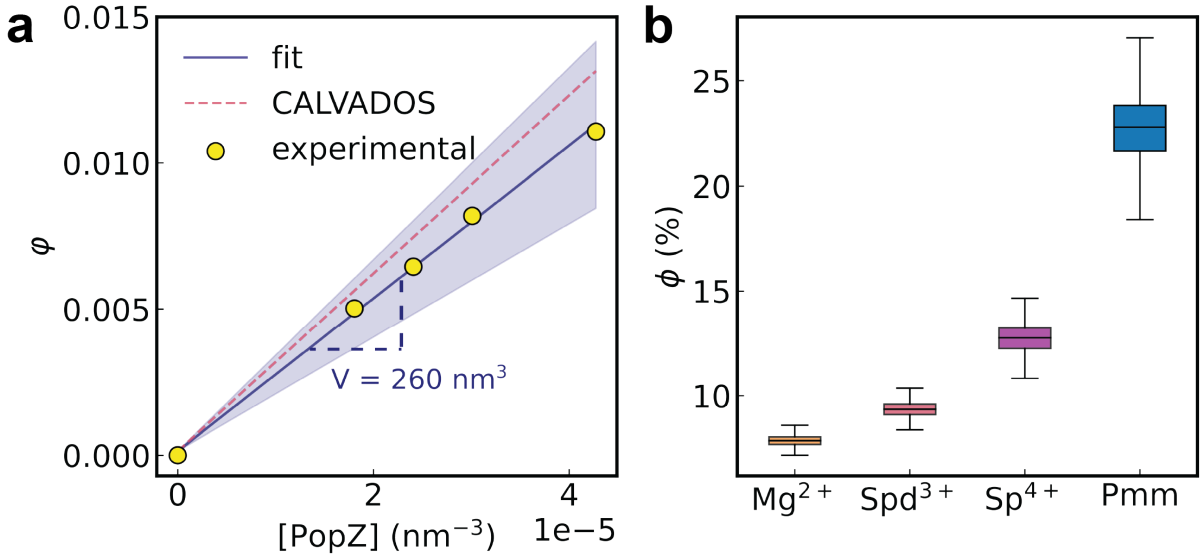

Figure S3: **Concentration and volume fraction measurements of PopZ condensates.** (a) Plot of calculated volume fractions ϕ as a function of the concentration of PopZ chains determined experimentally by measuring the index of refraction of PopZ monomer solutions of known concentrations using an Abbe refractometer. The blue shaded area shows the uncertainty in our estimation of the volume. These experimental results are consistent with estimates from CALVADOS (dashed line). The slope denotes the calculated volume, V of a single PopZ chain. (b) Measured volume fractions of condensates formed using different excipient multivalent ions.

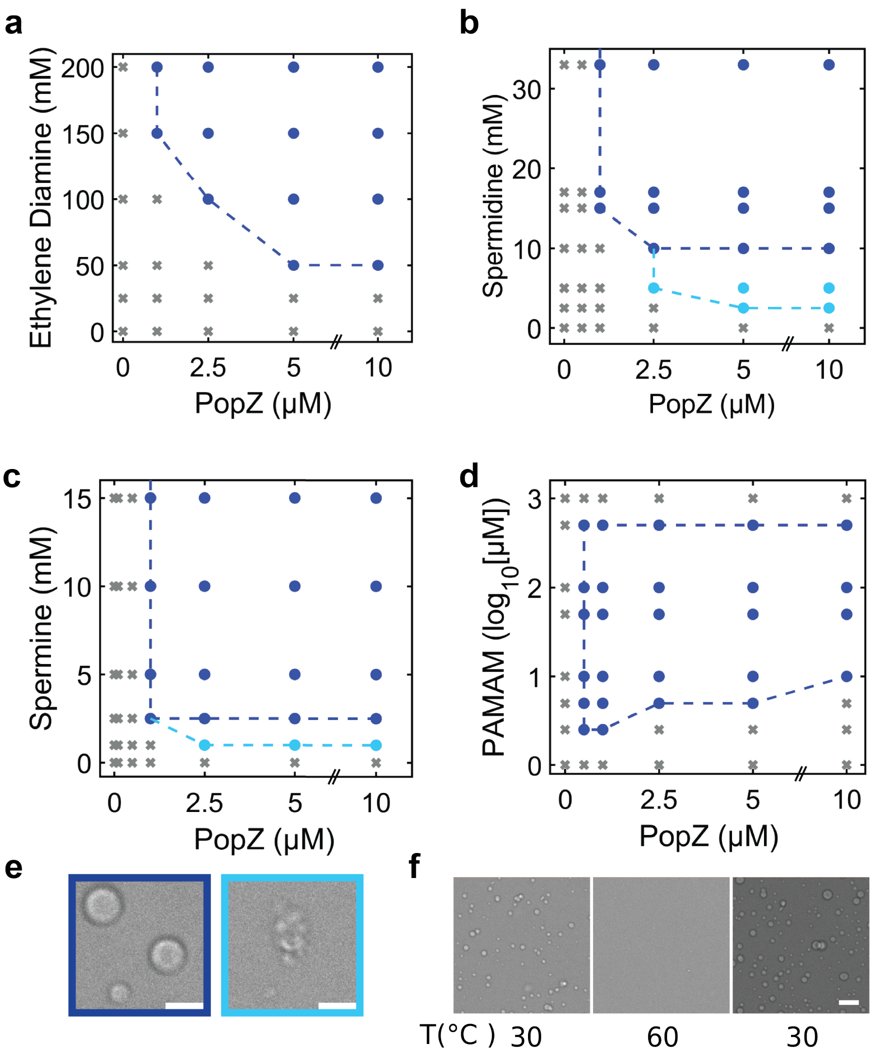

Figure S4: **Phase diagrams of PopZ in the presence of polyamines.** (a) Ethylene diamine ($EtDA^{2+}$), (b) Spermidine (Spd^{3+}), (c) Spermine (Sp^{4+}), and (d) PAMAM dendrimer (Pmm). Phase diagrams were measured using a multiwell imager, with PopZ reconstituted in 5 mM Sodium Phosphate (pH 7.0) and 10 mM NaCl. Data were collected using DIC microscopy. Gray X-marks indicate that no phase separation was observed. Spherical condensates were observed in conditions labeled with dark circles. Light blue circles indicate aspherical structures. Boundaries are drawn for reader's convenience. (e) Representative images of (left) a spherical condensate from 10 µM PopZ and 2.5 mM Sp^{4+}, and (right) an aspherical condensate from 10 µM PopZ and 1 mM Sp^{4+}. Scale bar: 5 µm. (f) DIC images of a PopZ condensate sample at 30 °C, after heating to 60 °C, and after cooling back to 30 °C in an imaging chamber. Scale bar: 10 µm.

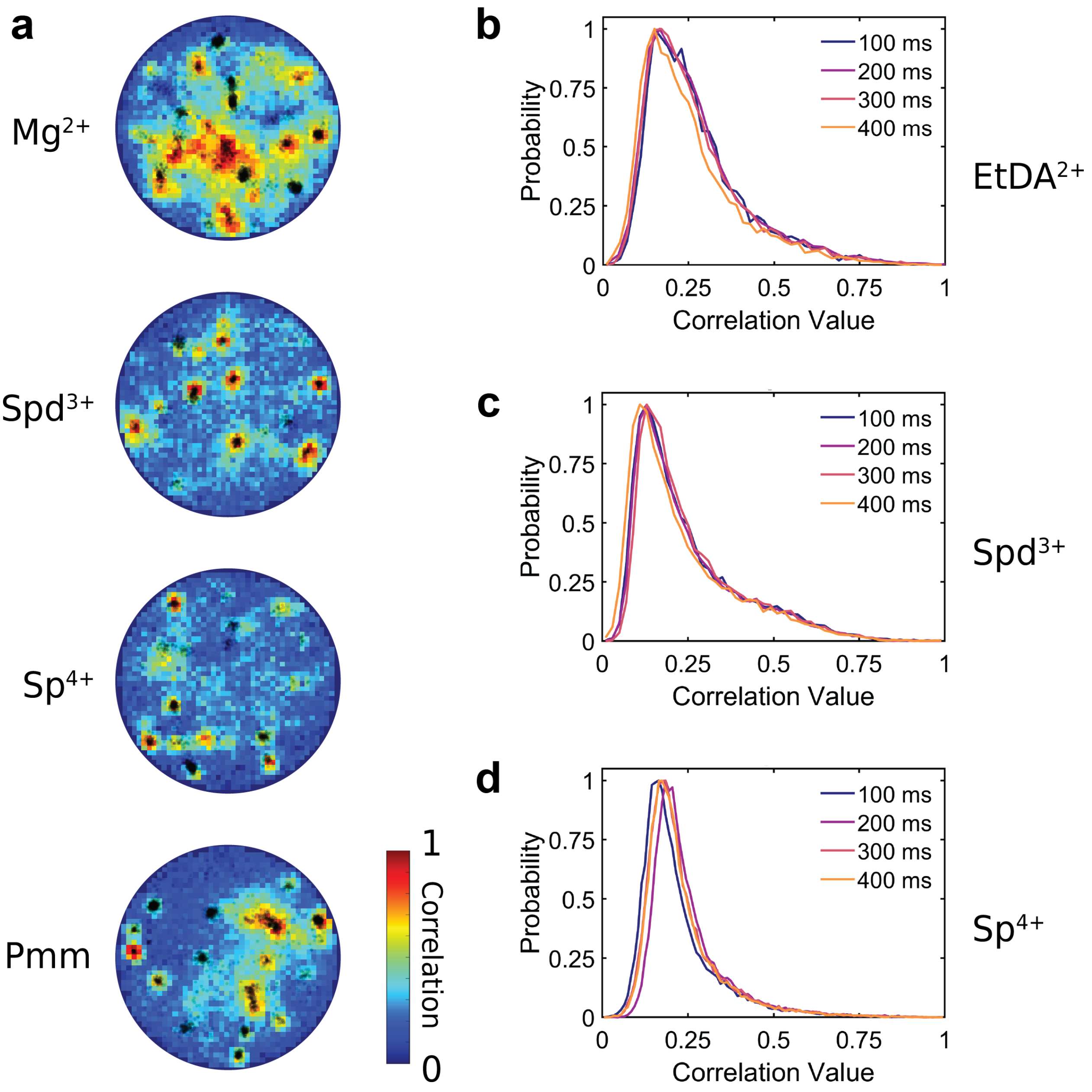

Figure S5: **Correlative single molecule localization and temporal image correlation analysis of PopZ condensates with various excipients.** (a) Single molecule localizations of dye-labeled PopZ shown in black superimposed on temporal image correlation analyses for representative condensates triggered by Mg^{2+}, Spd^{3+}, Sp^{4+}, and Pmm as described in Fig. 5a. Envelope histograms of correlation values for PopZ condensates triggered by (b) $EtDA^{2+}$, (c) Spd^{3+}, or (d) Sp^{4+} analyzed with time lags ranging in duration from 100 to 400 ms.

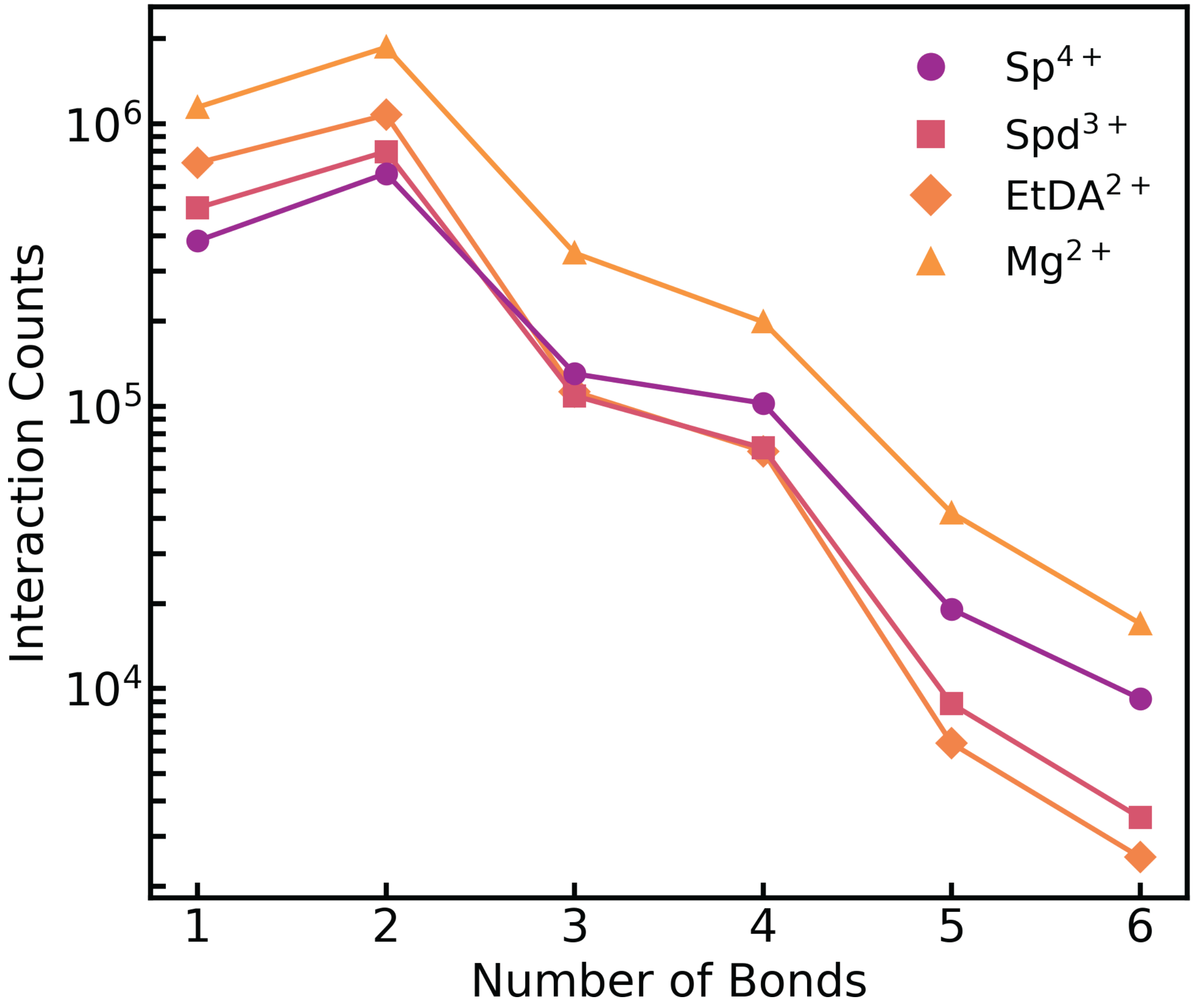

Figure S6: **Molecular interactions of PopZ with various excipients observed via MD simulation.** Total number of molecular interaction counts observed for each excipient ion with PopZ chain, for different number of bonds (ionic or hydrogen). These data are from the analysis of simulations run for a total time of 83.4 ns. Lines connecting the symbols are for reader's convenience.

Experimental

Protein Expression and Purification

PopZ, tagged with a His_6 epitope through a TEV protease cleavage site, was overexpressed in *E. coli* (BL21(DE3)) and purified under denaturing conditions.[S10,S11] Bacterial cells were lysed in a buffer containing 8 M urea, 500 mM NaCl, 50 mM sodium phosphate (pH 7.0), 10 mM imidazole, and 200 $mg\,mL^{-1}$ guanidinium chloride. His_6-tagged PopZ was purified using Ni-NTA resin beads in a gravity column. The purified protein was refolded via dialysis and stored at −80 °C in a buffer containing 50 mM sodium phosphate (pH 7.0), 100 mM NaCl, and 10 % glycerol (v/v) until further use.

Condensate Preparation

To prepare condensates, the His_6 tag was removed from PopZ by incubating the purified protein with 0.03 M/M TEV protease. The sample was subsequently dialyzed using a 3.5 kDa MWCO membrane for 1 h at room temperature into a final buffer of 5 mM sodium phosphate (pH 7.0) and 10 mM NaCl. Phase separation was induced by adding a multivalent cationic excipient—either $MgCl_2$, ethylene diamine, spermidine trichloride, spermine tetrachloride, or PAMAM—to the protein solution. The mixture was incubated at 30 °C for 1 h, unless otherwise stated.

Lipoic Acid Preparation and Addition

Lipoic acid solutions were prepared at 500 mM in 100 % DMSO and then diluted into 5 mM sodium phosphate (pH 7.0) and 10 mM NaCl to reach a final concentration of 50 mM. These solutions were further diluted into the condensate samples for a final lipoic acid concentration of 5 mM. During incubation, samples were mixed at 30 °C and 300 rpm on a standing shaker.

Differential Interference Contrast (DIC) Microscopy Sample Preparation

To minimize surface interactions, all well plates used for DIC microscopy were pre-treated with Tween-20. The wells were first flushed with N_2 gas to remove dust and debris, then coated with 1 % Tween-20 and incubated at 42 °C for 1 h. After incubation, the wells were washed five times with water at twice the volume of the Tween-20 solution. Finally, the wells were dried with N_2 gas and used within the same day.

Condensate Imaging with DIC

Differential Interference Contrast (DIC) microscopy was used to analyze phase boundaries and condensate size distributions. For phase boundary analysis, condensate samples were added to a Tween-20 treated well, sealed with an adherent film to prevent evaporation, and imaged using a multi-well plate imager (Cytation 5) equipped with a 60× air objective pre-heated to 30 °C. The use of a plate reader enabled rapid phase space imaging and phase boundary determination. Images were acquired approximately 15 min after adding the samples to the well plate.

For size distribution analysis, condensate samples were added to a Tween-20 treated well plate and imaged using an inverted microscope (Nikon Ti2) equipped with DIC optics, an oil immersion objective (Nikon PlanApo, 100×, 1.45 NA), and a sCMOS camera (Photometrics Prime 95B) with a system magnification of 0.11 µm/pixel. After 2 min of incubation, ten 1200 pixel × 1200 pixel Z-stacks were acquired with a 40 ms exposure time per frame for each sample within a 3 min time window.

For size distribution analysis, an optimal Z-height was determined for each region of interest (ROI) where condensates appeared with high contrast. Image segmentation was performed in MATLAB to estimate droplet diameters, while ensuring that out-of-focus condensates were excluded. Errors in diameter estimation were primarily due to variations in the

optimal Z-height for each droplet and uncertainty in droplet edge detection thresholds. Systematic offsets from potential substrate adhesion effects were not included in the uncertainty analysis.

For ground truth comparison of DIC and holography using silica beads, 1.57 µm Sphero™silica particles 5×10^{-5} % (w/v) dissolved in DI H_2O were added to a 384 well-plate and allowed to settle for 30 minutes. Sodium chloride (NaCl) was added to the well for a final concentration of 10 mM to promote sticking of silica particles onto the glass surface. Z-stacks were acquired with a 33 ms exposure time per frame. While the addition of NaCl reduced charge repulsion between neighboring silica particles, causing some aggregation, care was taken to image regions containing only monomeric particles. A representative image of monomeric silica particles imaged with DIC is shown in Fig. S1f. An optimal Z-height was determined for each ROI where silica particles appeared with high contrast as shown in Fig. S1f, and image segmentation was performed in MATLAB as described above.

Condensate Imaging and Characterization with Holographic Microscopy

Holographic imaging was performed using a commercial instrument (Spheryx xSight).[S1] 30 µL aliquots of condensate samples were transferred into a commercial microfluidic channel (Spheryx xCell8) with a height of 50 µm and a width of 500 µm. A pressure-driven Poiseuille flow transported the condensate particles through the channel at a maximum speed of $(3 \pm 1)\,\mathrm{mm\,s^{-1}}$. Dispersed particles entrained in the flow were illuminated by a 450 nm laser at an approximate intensity of $0.3\,\mathrm{mW/mm^2}$, and the resulting holograms were recorded.

The instrument analyzed each hologram using a generative model[S1,S12] based on the Lorenz-Mie theory of light scattering,[S13] extracting both the particle diameter, d_p, and refractive index, n_p. A single measurement on a micrometer-scale spherical particle yielded diameter estimates with a precision of ±2 nm and refractive index measurements accurate to within $\pm 1 \times 10^{-3}$.[S1,S12,S14] At particle concentrations around 10^7 particles/mL, statisti-

cal sampling of thousands of particles could be achieved and analyzed in under 15 min. The pipeline for holographic imaging and automated analysis is depicted schematically in Fig. 1b.

To measure silica beads using holography, 1.57 µm Sphero™silica particles 5×10^{-4} % (w/v) dissolved in DI H_2O were added to the microfluidic channel and analyzed as described above. It was previously described that particle dimers show a signature in holographic imaging greater in size and lower in refractive index than monomeric particles .[S15] Therefore, sizes were filtered to plot particles below 1.7 µm to visualize distributions of only monomeric silica particles (Fig. S1e).

Dye Labeling and Confocal Imaging of Condensates

PopZ-TEV-His_6 was labeled using either BODIPY-FL-NHS or JF646-SE dyes. PopZ-His_6 and the specific NHS-dye were incubated together in 5 mM sodium phosphate (pH 7.0) and 10 mM sodium chloride. The reaction mixture was kept in the dark with slow mixing for 6 h, followed by dialysis into 50 mM sodium phosphate (pH 7.0), 100 mM sodium chloride, and 10 % glycerol (v/v). Protein concentration was determined using the Pierce™ Bradford Plus Reagent, while dye concentration was determined from the absorbance spectrum of the solution. This conjugation protocol yielded labeled protein samples with a degree of labeling of 0.8 to 1 dye molecule per protein. The dye-labeled protein was flash-frozen and stored at −80 °C.

For dye-labeled protein condensate reconstitution, labeled PopZ was mixed with unlabeled protein at specific volume percentages. Samples were prepared as described in the section for DIC imaging. To measure relative fluorescence intensities of condensates, samples were added to a Tween-20 treated well plate and imaged after 5 min of incubation to allow for settling. Imaging was performed using a confocal laser scanning microscope (Abberior), and at least three 80 µm × 80 µm ROIs were collected per condition. Images were processed using bespoke Python programs. Thresholding was applied to isolate condensates, and the mean fluorescence intensity inside each condensate (F_{in}) was recorded. To deter-

mine background fluorescence, pixels inside the condensates were set to NaN, and the mean intensity of the remaining image was computed as F_{out}. The fluorescence ratio, $F_{\mathrm{in}}/F_{\mathrm{out}}$, was calculated for each condition, as shown in Fig. S3.

To determine relaxation times, 5 % (v/v) JF646-labeled PopZ was added to unlabeled PopZ in a 5 mM sodium phosphate (pH 7.0) and 10 mM sodium chloride buffer to achieve a final protein concentration of 5 µM. Phase separation was induced with 150 mM magnesium chloride. After a 1 h incubation at 30 °C, condensates were added to a Tween-20 treated well plate and imaged immediately using the confocal laser scanning microscope (Abberior)in the x-z plane. Five image sequences of 100 frames over 200 s were collected for ROIs measuring 70 µm to 90 µm (x-axis) by 10 µm to 14 µm (z-axis).

Single Molecule Localization Microscopy of PopZ Condensates

PopZ labeled with JF646 by NHS-ester conjugation as previously described was added to an unlabeled sample of PopZ at 0.001 % v/v. After condensate formation, samples were added to a Tween-20 treated 384-well glass-bottom plates (number 1.5 , Sigma). The condensates attached on the surface of the well were imaged within 10 minutes after addition to the well plate. 1000 frames were collected at an exposure time of 20 ms for each ROI. All samples were imaged under identical illumination and acquisition settings to allow for direct comparison of localization densities. While no explicit correction was applied for fluorophore blinking or repeated localizations, the low labeling density and moderate excitation power were selected to minimize these effects, enabling reliable relative quantification across conditions. At least six 256 × 256 pixel ROIs were collected for each sample. The number of condensates analyzed in each case were: Mg^{2+} (23), $EtDA^{2+}$ (10), Spd^{3+} (16), Sp^{4+} (20), and Pmm (31).

ThunderSTORM[S16] analysis with normalized Gaussian visualization from FIJI was performed on representative condensates for each sample, as shown in Fig. 5. These localizations were then clustered using DBScan in MATLAB[S17] with a neighborhood search radius, epsilon, of 150 nm and 15 minimum neighbors to plot the clustered single molecule localizations

shown in Fig. 5a. For temporal image correlation spectroscopy (TICS), condensate images were first thresholded to include intensity values from pixels only within the condensate using a threshold T calculated by the following:

$$T = \langle I_{bkg} \rangle + 3\sigma_{bkg}, \tag{11}$$

where the mean intensity $\langle I_{bkg} \rangle$ and standard deviation σ_{bkg} of the background were used. Intensities at each pixel of a video were normalized to z scores using the following equation,

$$z(x, y, c) = \frac{I(x, y, c) - \langle I(x, y) \rangle}{\sigma(x, y)}, \tag{12}$$

where $z(x, y, c)$ represents the z score at a specific frame number c and pixel x, y. $I(x, y, c)$ represents the raw intensity value at the frame number c and $\langle I(x, y) \rangle$ is the average intensity at that pixel. $\sigma(x, y)$ is the standard deviation of intensity values at that pixel. These z scores were then processed further to determine the average correlation value at that pixel with a specified time lag τ with units in frames:

$$\langle r(x, y) \rangle = \frac{1}{N} \sum_{c=0}^{N} z(x, y, c)\, z(x, y, c + \tau), \tag{13}$$

where N denotes the the total number of frames collected for each video after subtracting the timelag τ. This process was performed for all pixels within a condensate using a lag time of 1 frame (20 ms) unless otherwise stated, and the average correlation values per pixel were plotted either as a heat map or enveloped histogram. To account for photobleaching, a correction factor was calculated based on previous calculations using TICS.[S18] Briefly, average intensities of the condensates of a single field of view were plotted as a function of time t. The intensities were normalized and fit to an exponential decay curve,

$$y = A\, e^{-kt}, \tag{14}$$

where y represents the normalized average intensity and k is the bleaching decay constant. Average correlation values were then multiplied by the correction factor,

$$cf = \frac{e^{k(N-\tau)} - 1}{k(N-\tau)}, \tag{15}$$

to produce the final correlation values, which were normalized and spatially resolved as in Fig. 5.

All-atom Molecular Dynamics Simulations of PopZ and Multivalent Cations

All-atom MD simulations were performed to capture details of molecular interactions between PopZ and multivalent ions.The simulation system was built by randomly placing 14 chains of PopZ protein in a 20 nm cubic box, which was subsequently solvated by TIP4P water.[S19] Keeping ionic strength of 2 M, multivalent ions, including Mg^{2+}, $EtDA^{2+}$, Spd^{3+}, and Sp^{4+}, were introduced into the box to mimic the experimental condition, while chloride ions were added to neutralize the system. The Mg^{2+} box contained 242 324 water molecules, 3279 Mg^{2+} ions, and 6152 Cl^{-} ions. The $EtDA^{2+}$ box included 229 256 water molecules, 3279 $EtDA^{2+}$ molecules, and 6152 Cl^{-} ions. The Spd^{3+} box was composed of 230 125 water molecules, 1640 Spd^{3+} molecules, and 4514 Cl^{-} ions. Finally, the Sp^{4+} box consisted of 234 521 water molecules, 984 Sp^{4+} molecules, and 3530 Cl^{-} ions. Periodic boundary conditions were applied in all three dimensions for all systems. Energy minimization was performed using the steepest descent algorithm followed by 100-ps equilibration, with a step size of 2 fs, under NVT conditions at 300 K. Protein position restraints were applied during NVT run. Further NPT equilibration was performed for 100 ps with a 2-fs time step, using the Parrinello-Rahman barostat for pressure coupling to maintain a pressure of 1 bar, without any restraints on protein position. The production MD runs were carried out for 83.4 ns, with a time step of 2 fs. Energy and compressed coordinate data were saved every 10 ps (5000 steps) for

subsequent analysis. All simulations were conducted using GROMACS 2023.3[S20] and the AMBER99SB force field.[S21]

Hydrogen bonds between negative residues on PopZ chains and multivalent cations were identified using a cutoff distance of 3.9 Å between acceptors and donors. Ionic bonds between negative residues on PopZ chains and Mg^{2+} ions were defined using a cutoff distance of 5 Å.[S22] Each frame of the trajectory file was analyzed, looping over all multivalent ions to identify interactions and quantify the number of protein interactions per ion. The total number of frames where a certain number of interactions, 1 to 6, was counted and presented in a logarithmic-scale plot with the number of interactions on the x-axis and the log of total number of interaction counts on the y-axis. By analyzing interaction modes and the total number of interaction counts, we predict the effect of multivalent ions on the stability of the protein system. Notably, the short and long runs exhibited similar trends, reinforcing the consistency of the observed dynamics.

Acknowledgement

The authors thank members of the Grier and Saurabh research groups, and Prof. Alexander Grosberg (NYU) for helpful discussions and inputs. This study was supported by National Institutes of Health through award 1R35GM157103 to SS, and by the National Science Foundation (NSF) through award DMR-2104837 to DGG. SW was supported by an NSF funded REU site in chemical biology at NYU Chemistry. The xSight instrument used for this study was acquired as shared instrumentation with support from the MRSEC program of the NSF under award DMR-1420073.

References

(S1) Lee, S.-H.; Roichman, Y.; Yi, G.-R.; Kim, S.-H.; Yang, S.-M.; Van Blaaderen, A.; Van Oostrum, P.; Grier, D. G. Characterizing and tracking single colloidal particles

with video holographic microscopy. *Opt. Express* **2007**, *15*, 18275–18282.

(S2) Odete, M. A.; Cheong, F. C.; Winters, A.; Elliott, J. J.; Philips, L. A.; Grier, D. G. The role of the medium in the effective-sphere interpretation of holographic particle characterization data. *Soft Matter* **2020**, *16*, 891–898.

(S3) Markel, V. Introduction to the Maxwell Garnett approximation: Tutorial. *J. Opt. Soc. Am. A* **2016**, *33*, 1244–1256.

(S4) Cheong, F. C.; Xiao, K.; Pine, D. J.; Grier, D. G. Holographic characterization of individual colloidal spheres' porosities. *Soft Matter* **2011**, *7*, 6816–6819.

(S5) McMeekin, T. L.; Groves, M. L.; Hipp, N. J. In *Amino Acids and Serum Proteins*; Stekol, J. A., Ed.; American Chemical Society: Washington, DC, 1964; Vol. 44; Chapter 4, pp 54–66.

(S6) Zhao, H.; Brown, P. H.; Schuck, P. On the distribution of protein refractive index increments. *Biophys. J.* **2011**, *100*, 2309–2317.

(S7) Tesei, G.; Schulze, T. K.; Crehuet, R.; Lindorff-Larsen, K. Accurate model of liquid–liquid phase behavior of intrinsically disordered proteins from optimization of single-chain properties. *Proc. Natl. Acad. Sci.* **2021**, *118*, e2111696118.

(S8) Lin, J. Divergence measures based on the Shannon entropy. *IEEE Trans. Inf. Theory* **1991**, *37*, 145–151.

(S9) Székely, G. J.; Maria, R. L.; Nail, B. K. Measuring and Testing Dependence by Correlation of Distances. *Ann. Stat.* **2007**, *35*, 2769–2794.

(S10) Bowman, G. R.; Perez, A. M.; Ptacin, J. L.; Ighodaro, E.; Folta-Stogniew, E.; Comolli, L. R.; Shapiro, L. Oligomerization and higher-order assembly contribute to sub-cellular localization of a bacterial scaffold. *Mol. Microbiol.* **2013**, *90*, 776–795.

(S11) Saurabh, S.; Chong, T. N.; Bayas, C.; Dahlberg, P. D.; Cartwright, H. N.; Moerner, W. E.; Shapiro, L. ATP-responsive biomolecular condensates tune bacterial kinase signaling. *Sci. Adv.* **2022**, *8*, eabm6570.

(S12) Martin, C.; Altman, L. E.; Rawat, S.; Wang, A.; Grier, D. G.; Manoharan, V. N. In-line holographic microscopy with model-based analysis. *Nat. Rev. Methods Primers* **2022**, *2*, 83.

(S13) Bohren, C. F.; Huffman, D. R. *Absorption and Scattering of Light by Small Particles*; Wiley Interscience: New York, 1983.

(S14) Krishnatreya, B. J.; Colen-Landy, A.; Hasebe, P.; Bell, B. A.; Jones, J. R.; Sunda-Meya, A.; Grier, D. G. Measuring Boltzmann's constant through holographic video microscopy of a single colloidal sphere. *Am. J. Phys.* **2014**, *82*, 23–31.

(S15) Altman, L. E.; Quddus, R.; Cheong, F. C.; Grier, D. G. Holographic characterization and tracking of colloidal dimers in the effective-sphere approximation. *Soft Matter* **2021**, *17*, 2695–2703.

(S16) Ovesný, M.; Křížek, P.; Borkovec, J.; Švindrych, Z.; Hagen, G. M. ThunderSTORM: a comprehensive ImageJ plug-in for PALM and STORM data analysis and super-resolution imaging. *Bioinformatics* **2014**, *30*, 2389–2390.

(S17) Ester, M.; Kriegel, H.-P.; Xu, X. A Density-Based Algorithm for Discovering Clusters in Large Spatial Databases with Noise. Proceedings of the Second International Conference on Knowledge Discovery and Data Mining, KDD'96. AAAI Press. **1996**.

(S18) Kolin, D. L.; Costantino, S.; Wiseman, P. W. Sampling Effects, Noise, and Photobleaching in Temporal Image Correlation Spectroscopy. *Biophys. J.* **2006**, *90*, 628–639.

(S19) Jorgensen, W. L.; Chandrasekhar, J.; Madura, J. D.; Impey, R. W.; Klein, M. L. Comparison of simple potential functions for simulating liquid water. *J. Chem. Phys.* **1983**, *79*, 926–935.

(S20) Abraham, M. J.; Murtola, T.; Schulz, R.; Páll, S.; Smith, J. C.; Hess, B.; Lindahl, E. GROMACS: High performance molecular simulations through multi-level parallelism from laptops to supercomputers. *SoftwareX* **2015**, *1*, 19–25.

(S21) Ponder, J. W.; Case, D. A. Force fields for protein simulations. *Adv. Protein Chem.* **2003**, *66*, 27–85.

(S22) Tam, J. Z.; Palumbo, T.; Miwa, J. M.; Chen, B. Y. Analysis of Protein-Protein Interactions for Intermolecular Bond Prediction. *Molecules* **2022**, *27*, 6178.
